# Long-Term Passive Leg Stretch Improves Systemic Vascular Responsiveness as Much as Single-Leg Exercise Training

**DOI:** 10.1249/MSS.0000000000002811

**Published:** 2021-10-25

**Authors:** EMILIANO CÈ, MASSIMO VENTURELLI, ANGELA VALENTINA BISCONTI, STEFANO LONGO, ANNA PEDRINOLLA, GIUSEPPE CORATELLA, FEDERICO SCHENA, FABIO ESPOSITO

**Affiliations:** 1 Department of Biomedical Sciences for Health, Università degli Studi di Milano, Milan, ITALY; 2 IRCCS Istituto Ortopedico Galeazzi, Milan, ITALY; 3 Section of Movement Science, Department of Neuroscience, Biomedicine, and Movement Science, University of Verona, Verona, ITALY; 4 Section of Geriatrics, Department of Internal Medicine, University of Utah, Salt Lake City, UT

**Keywords:** FLOW-MEDIATED DILATION, SHEAR RATE, SINGLE-LEG KNEE EXTENSION TRAINING, SINGLE PASSIVE LIMB MOVEMENT, VASCULAR CONDITIONING, VASCULAR PLASTICITY

## Abstract

**Purpose:**

The current study compared the local and systemic vascular responsiveness after small muscle mass endurance training or passive stretching training (PST).

**Methods:**

Thirty-six sex-matched healthy participants underwent 8-wk single-leg knee extension (SLKE) (*n* = 12) training or PST (*n* = 12), or no intervention (control, *n* = 12). Before and after the intervention, local and systemic vascular responsiveness was assessed by Doppler ultrasound at the femoral (local effect) and brachial artery (systemic effect) during single passive leg movement and brachial flow-mediated dilation (FMD) test, respectively.

**Results:**

After training, delta femoral blood flow (representing the local vascular responsiveness) increased after SLKE and PST by +54 (7)% (effect size, 2.72; *P* < 0.001) and +20 (2)% (effect size, 2.43; *P* < 0.001), respectively, albeit with a greater extent in SLKE (post-SLKE vs post-PST: +56 [8]% [effect size, 2.92; *P* < 0.001]). Interestingly, the %FMD (standing for the systemic effect) increased after SLKE and PST by +12 (2)% (effect size, 0.68; P < 0.001) and +11 (1)% (effect size, 0.83; *P* < 0.001), respectively, without any between-groups difference (*P* > 0.05). No changes occurred in control.

**Conclusions:**

The present findings revealed that both active and passive training modalities induced similar improvements in the brachial artery dilatation capacity, whereas the former was more effective in improving femoral artery blood flow. Passive stretching could be used in people with limited mobility to improve vascular responsiveness both at the local and systemic level and in this latter case has similar effects as small muscle mass endurance training.

## Abstract 2

Supplemental digital content is available in the text.

Vascular responsiveness is the ability of the arterial tree to dilate and constrict and represents an important marker of cardiovascular health and a predictor of cardiovascular diseases (1–4). Vascular responsiveness decreases with advancing age and is compromised in people with heart disease, metabolic alterations, or limited mobility (5–10), determining a further increase in the cardiovascular risk in these populations. Indeed, alterations in vascular responsiveness often precede an increase in arterial stiffness, a prognostic index of cardiovascular health (11). Given its importance, numerous studies investigated how to ameliorate vascular responsiveness through both pharmacological and nonpharmacological approaches (12–16). The nonpharmacological approach often involves endurance training performed with large muscle mass (e.g., running, jogging, or cycling) and has received the most attention and endorsement in terms of effectiveness in improving vascular responsiveness (12–15).

In recent years, endurance training involving small muscle mass, such as single-leg knee extension training (SLKE), has also received growing interest as a method for improving vascular responsiveness. Single-leg knee extension training is being used to maximize vascular adaptation because of its ability to circumvent central limitations and sympathetic restraint of limb blood flow (*Q*˙) (17). Endurance exercise using small muscle mass was also shown to improve local circulation, oxygen extraction, and muscle force (18–20). The increased metabolic demand of the muscles involved, such as a higher oxygen requirement from the contracting muscles and the need to remove some exercise-induced metabolites, matches the increment in *Q*˙ and therefore in shear rate (*Y*˙), the frictional force acting on the vessel’s inner lumen (i.e., the mechanical stress acting on the endothelial cells) (21–23). In addition, although with conflicting results (18), a systemic effect on vascular responsiveness (i.e., on the arteries not directly involved in the exercise) was also reported (22). Despite its greater feasibility compared with endurance training, small muscle mass endurance exercise might still remain precluded to people with limited mobility.

On this path, an increasing body of literature has focused on the effects of passive exercise methods (e.g., passive static stretching) as a means to maintain or even improve vascular responsiveness, reporting very interesting and encouraging results (24,25). For example, 60 sessions of passive static stretching performed over 12 wk significantly improved vascular responsiveness at both local and systemic levels (24). A plausible explanation for the effects of passive static stretching at the local level may depend on the repetitive increases and decreases in *Y*˙ occurring during the elongation and relaxation phases of passive static stretching, which can trigger a chain of reactions that may lead to higher endothelial nitric oxide (NO) bioavailability, thus increasing the vascular responsiveness (9,18,26). At the systemic level, the continuous stretch-induced fluctuations in *Y*˙ could generate a continuative alternance of vasoconstriction and vasodilation, and consequently a possible stimulus toward the autonomic nervous system in controlling the *Q*˙ distribution (11,24,27).

Although not uniquely reported in the literature, the fluctuations in *Y*˙ may also play an important role during endurance small muscle mass exercise as the SLKE (18). Although the mechanism might be similar, the extent of its effects on both local and systemic vascular responsiveness when performing SLKE or passive static stretching training (PST) was assessed in separate studies and has not been directly compared in the same population. Should the results be comparable, people with limited mobility may benefit from performing PST, definitely less demanding and more feasible than SLKE. On these bases, the purpose of the present study was to evaluate the effects of SLKE versus PST on the vascular responsiveness at both local and systemic levels determined by Doppler ultrasound using the single passive limb movement (sPLM) and flow-mediated dilation (FMD) test. It was hypothesized that both SLKE and PST would improve vascular responsiveness, with the former inducing greater adaptations.

## METHODS

### Participants

**FIGURE 1:**
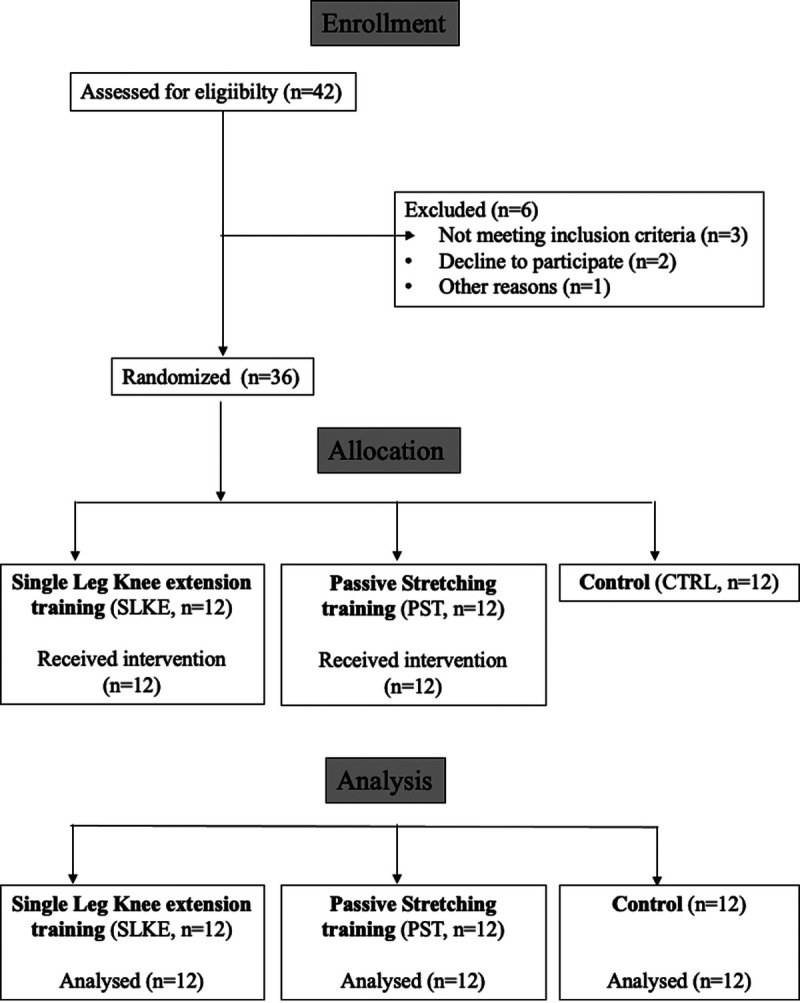
Study flowchart.

Figure 1 illustrates the study flowchart. A total of 42 low-active healthy adults volunteered to participate in the study (age range, from 20 to 30 yr). The level of physical activity was determined by the Italian version of the International Physical Activity Questionnaire (28). Exclusion criteria were as follows: (i) presence of neurological, vascular, and musculoskeletal impairments at the level of the lower and upper limbs; (ii) being on pharmacological therapy related to neural and/or vascular response, including hormonal contraceptives and oral supplements; (iii) being a current or former smoker; (iv) systolic arterial pressure higher than 140 mm Hg and diastolic higher than 90 mm Hg; and (v) having an irregular menstrual cycle (shorter than 26 d or longer than 35 d) up to 3 months before the beginning of the study; (vi) contraindications to joint mobilization; and (vii) being regularly involved in aerobic or PST program. All participants supplied written, informed consent after being informed about the aims of the study and the potential risks derived from tests and methods. The institutional review board of the Università degli Studi di Milano approved the study (CE 27/17). The study was registered at ClinicalTrial.gov (ID: NCT04758754) and performed by the principles of the Helsinki Declaration. The researchers who analyzed the data were blinded to group allocation.

### Study Design

Before testing procedures, participants underwent a preliminary session during which they familiarized themselves with the dynamic knee extension ergometer and with the procedure to identify the maximum isometric voluntary contraction (MVC) of the knee extensor muscles of both limbs. During this visit, the sPLM and the FMD tests (see below for a full explanation of the procedures) were performed on each participant. At the end of the tests, the ultrasound probe position for testing was marked on a transparency sheet, together with some skin landmarks (moles, scars, angiomas, etc.). The sPLM and FMD outcomes obtained during the familiarization and the pretraining experimental session were used to calculate intersession reliability. Knee flexion range of motion (ROM) was also measured.

All measurements were taken at the beginning (PRE) and after 8 wk (POST) of SLKE or PST training. The participants were tested at the same time of the day in a climate-controlled laboratory (temperature 20°C [1°C] and relative humidity 50% [5%]) to minimize confounders due to circadian rhythms. For females, the tests were assessed on the same day of the menstrual cycle [early follicular phase day 3 (3)]. Female participants recorded their menstrual cycle in a personal diary throughout the study, which was used to assess the early follicular phase and allowed the subjects to be tested on the same menstrual day pretraining and posttraining. On the test days, participants came to the laboratory after fasting overnight, abstaining from caffeine and other similar substances for at least 12 h and not taking part in heavy exercise for at least 48 h before the tests. Posttraining assessments were performed 48 h after the last training session. As in a previous study, this period was observed to possibly prevent biases in measurements introduced by the acute effects of the last training session (18).

### Measurements and Data Analysis

Measurements were performed bilaterally in all groups for the lower and upper limbs. Data are presented as the average between the two limbs.

#### Range of motion

To check the changes in knee joint ROM, a bi-axial angle transducer (TSD 130A; Biopac System, Goleta, CA) was used. The angle transducer was placed with one axis on the external condyles of the knee joint and the other on the external face of the fibula.

#### Maximum isometric voluntary contraction

Knee extensors MVC was measured with the participants sitting on an ergometer with the hip and the knee flexed at 90° and firmly secured at the ankle level by a Velcro® strap (Velcro Industries Inc., Willemstad, Netherlands Antilles) to a load cell (mod. SM-2000N operating linearly between 0 and 2000 N; Interface, Crowthorne, United Kingdom) for the force signal detection. After a standardized warm-up (10 × 2-s contractions at 50% MVC previously determined during familiarization), three MVC attempts were performed. The participants were instructed to push as fast and hard as possible for 3 s. Each MVC attempt was interspersed by 3 min of recovery. The force signal was driven to an A/D converter (mod. UM 150, Biopac, Biopac System Inc., Santa Barbara, CA), sampled at 1000 Hz, and stored on a personal computer. The maximum force value recorded during tests was considered MVC and was inserted into the data analysis.

#### Maximum work rate

The maximum work rate (W˙_max_) was determined by an incremental square wave test on a dynamic knee extension ergometer. The test was performed while sitting on an adjustable chair to fit body sizes of different dimensions. Both knees were flexed at 90°, with the ankle of the dominant limb connected to a bicycle ergometer pedal arm by a rigid bar. The concentric phase occurred actively from 90° of the knee to full extension, whereas the eccentric phase was completely passive, driven by the flywheel momentum. The mechanical brake applied to the ergometer and the pedal frequency was measured to determine the mechanical power output. The mechanical friction, that is, the force applied to each revolution, was measured by a force transducer (mod. SM-100N; Interface), while the pedal frequency was determined by a magnetic transducer integrated into the cycle ergometer (mod. 839E; Monark, Vansbro, Sweden). Both the force and the pedal frequency signals were amplified (gain × 100) and acquired by a personal computer after an A/D conversion (model UM150; Biopac System) at a sampling rate of 1 kHz. The square wave test started with a first workload of 15 W. Loads increased by +5 W until exhaustion (29). Each load was kept for 3 min, with 5 min recovery in between. The load of the last completed step was considered as W˙_max_. A noninvasive impedance cardiograph device (Physio Flow®; Manatec Biomedical, Paris, France) was used to assess the cardiac output (*Q*˙_T_), stroke volume (q), and HR. At rest and during the test, pulmonary oxygen uptake (V˙O_2_) was detected on a breath-by-breath modality by gas analyzers (mod. Quark b^2^, Cosmed, Rome, Italy). The system was calibrated before each test with gas mixtures of known concentrations (16% O_2_; 5% CO_2_; balance N_2_). Data were averaged on the last 60 s of baseline and the last 30 s of the last workload. The average V˙O_2_ matched to W˙_max_ was considered as peak V˙O_2_ (V˙O_2_p).

#### Muscle thickness

Participants lay in a supine position on an examination bed with their knees in the anatomical position. By real-time B-mode ultrasonography with a 9-MHz linear (mod. Logiq-7; General Electric Medical Systems, Milwaukee, WI), three images were taken on the center of vastus lateralis, vastus intermedius, and rectus femoris muscle belly using sagittal plane ultrasound scans, halfway between the lateral epicondyle of the knee and the greater trochanter. The site was clearly marked on the skin with indelible ink and further outlined on tracing paper, to supply a standardized measurement site and ensure that assessments at all measurement points were taken from the same external site. Minimal pressure was applied on the skin during scans, which were saved and analyzed offline with publicly available imaging software (ImageJ 1.43b; NIH, Bethesda, MD). Muscle thickness, defined as the perpendicular distance between the superficial and deep aponeurosis, was measured in the proximal, mid, and distal regions in each ultrasound image, and the average of the three values was used for further analyses.

#### Single passive limb movement

Single passive limb movement was performed by the recommended procedures (30). Sitting on a chair, subjects rested in the upright-seated position for 10 min before starting the data collection and remained in this position until the end of the test. The sPLM protocol consisted of 60 s of baseline peripheral hemodynamic data collection, followed by single passive knee flexion and extension of 1 s, after which the leg was kept fully extended for the remaining 59 s for the postmovement data collection. sPLM was performed by a member of the research team, who moved the subject’s lower leg through a 90° ROM at 1 Hz. Throughout the test, measurements of arterial blood velocity and vessel diameter were performed in the common femoral artery of the passively moved leg, distal to the inguinal ligament and proximal to the deep and superficial femoral bifurcation by Doppler ultrasound (mod. Logiq-7; General Electric Medical Systems). After being positioned to an insonation angle of 60° or less, a 9-MHz linear array transducer was used to measure the mean blood velocity. The sample volume was centered and size-maximized according to the vessel’s diameter (31). Femoral artery *Q*˙ (*Q*˙_fem_) was calculated by using data of arterial diameter and mean blood velocity as:

*Q*˙ (mL·min^−1^) = mean blood velocity × π × (diameter / 2)^2^ × 60

The cumulative *Q*˙_fem_ was integrated (area under the curve [AUC]) using the trapezoidal rule and then calculated. Reliability analysis for sPLM was previously reported (18,24).

Before the sPLM, the systolic and diastolic arterial pressure was measured at rest by a digital sphygmomanometer (mod. HEM-907; Omron, Hoofddorp, The Netherlands). The mean arterial pressure (MAP) was therefore calculated.

#### Flow-mediated dilatation

Flow-mediated dilation was measured at the brachial artery according to the recommended procedures (32). Before FMD, the participants lay supine for 20 min to restore baseline cardiovascular values. An arterial pressure cuff was placed on the forearm immediately distal to the olecranon process to generate an ischemic stimulus in the brachial artery when inflated. Following baseline assessment, the blood pressure cuff was inflated to 250 mm Hg for 5 min. Arterial diameter and mean velocity recordings resumed at baseline, 30 s before cuff deflation, and continued for 120 s postdeflation, as described elsewhere (10,19,32). A 15-MHz linear array transducer attached to a high-resolution ultrasound machine was used to image the brachial artery in the distal third of the upper arm. When an optimal image was obtained, the probe was held stable and longitudinal in B-mode, and images of the lumen-arterial wall interface were acquired. Continuous Doppler velocity was measured simultaneously, and the data were collected using the lowest possible insonation angle (<60°). The FMD data were exported in AVI format and analyzed using commercially available software (Brachial Artery Analyzer for Research; Medical Imaging Applications, LLC, Coralville, IA). An experienced operator double-checked the analysis of the data. Flow-mediated dilation was quantified as the maximal change in an artery diameter after cuff release, expressed as a percentage increase above baseline (%FMD):


$$\text{\%}\text{FMD}=\left(\text{peak diameter}-\text{baseline diameter}\right)/\text{baseline diameter}\times 100.$$

Brachial artery blood flow (*Q*˙_brac_) was calculated as described for sPLM measurement.

Brachial artery *Y*˙ was calculated postcuff release with the equation:


$$\overset{\cdot }{Y}\;\left(s^{-1}\right)=8\;\text{mean velocity}/\text{diameter}$$

The cumulative *Y*˙, corresponding to the reactive hyperemia postcuff release (total *Y*˙ from cuff release to time-to-peak, $\overset{\cdot }{Y}$ AUC) was calculated using the trapezoidal rule. The *Y*˙ AUC reflects the amount of mechanical stimulus applied to the endothelium during cuff release hyperemic response until time to peak. Given that FMD is primarily dependent on endothelial response to mechanical stimuli, the FMD was divided by the *Y*˙ AUC (FMD/*Y*˙) (21). Reliability analysis for FMD was reported previously (18,24).

#### Acute hemodynamic measurements

**FIGURE 2:**
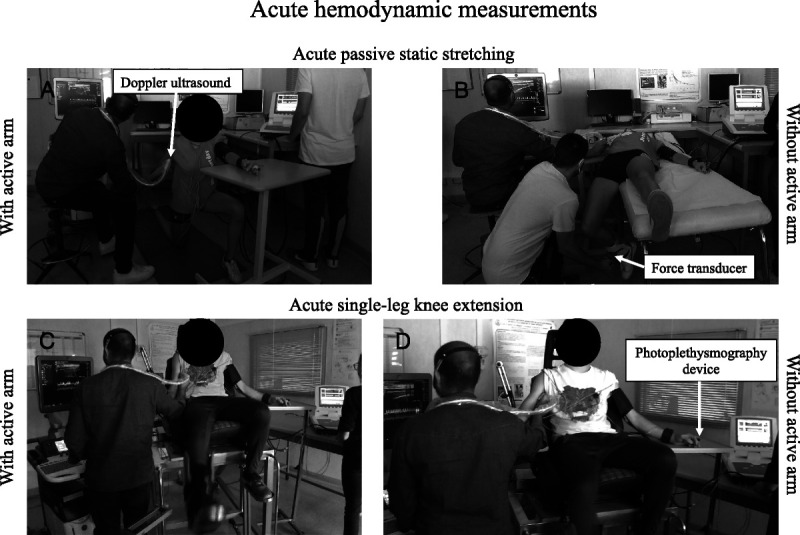
Acute hemodynamic measurements. Passive stretching bout. Acute passive stretching bout of the knee extensor muscles with the right arm maintaining actively the elongation and the left arm positioned on a table to allow the positioning of the finger photoplethysmography device without any constriction (panel A), acute passive stretching bout the knee extensor muscles stretched by an operator (panel B). SLKE. Exercise performed with (panel C) and without (panel D) the stabilization of the participant’s position on the ergometer.

To evaluate the possible difference in the brachial artery vascular response between the exercise conducted without the use of the upper limbs and that with the use of the upper limbs (a condition similar to that used during training), the *Y*˙ and the MAP were assessed in all the study participants (i) during an acute 5-min SLKE at 70% W˙_max_ (representing a typical workload intensity within the training) and (ii) during a single-bout PST of the knee extensor muscles, both performed with and without the use of the upper limb muscles (see Fig. 2 for a photographic representation of the experimental setup). These evaluations were performed in different sessions separated by at least 72 h. During the two acute evaluations, the artery’s diameter and the mean velocity were continuously measured from the brachial artery of the right arm, that is, the arm stabilizing the stretched muscle during PST administration and the participant’s position on the ergometer during SLKE. The mean *Y*˙ during the 5-min single-leg exercise and the five elongations and relaxation phases of the passive stretching bout was calculated as in the FMD tests (Doppler ultrasound, mod. Logiq-7; General Electric Medical Systems). Concomitantly, the MAP was determined on a beat-by-beat basis using a finger photoplethysmography device (FinometerPro; Finapres Medical Systems, Amsterdam, The Netherlands), with the photoplethysmography cuff placed on the third finger of the left hand. The height adjustment and reference sensors were positioned following the manufacturer’s instructions. The blood pressure signal was calibrated by the procedure indicated by the manufacturer.

The *Y*˙ and the MAP were calculated over time windows of 5 s and then averaged every minute. Only for the measurements relating to stretching, the *Y*˙ and the MAP were also calculated considering separately the elongation phases (average over 45 s) and the relaxation phase (average over 15 s), due to a different behavior that two variables arise during the two phases (24).

During the single-leg knee exercise, the participants sat on the same ergometer in the test for W˙_max_ determination. The assessment started with a 3-min measurement of baseline condition, then measurements were made continuous throughout the 5-min exercise (18). Exercise was performed with and without stabilizing the position of the participant on the ergometer.

During the passive stretching assessment, the participants rested in a supine position for 20 min before starting the data collection and remained in this position throughout the entire duration of the data collection (33). Passive stretching protocol consisted of 5 min of resting baseline followed by passive static elongations for 45 s and passive relaxations for 15 s, repeated five times. During the entire PST protocol, the muscles were stretched by the same operator up to a point of discomfort. The knee joint angle was continuously recorded using a biaxial angle transducer (mod: TSD 130A; Biopac Systems). Force output between the passively stretched leg and the operator’s arms was recorded during the protocol by a load cell (model SM-2000N; Interface). Specifically, the load cell was positioned 5 cm above the ankle, and a member of the research team pushed perpendicularly the load cell to stretch the leg extensor for 45 s. The passive force output during the 45 s of the consecutive flexion of the PS protocol was displayed on a computer screen to keep constant the passive force throughout the elongations. In the second session, the measurements were performed with the participant positioned as in Figure 2B, which is the stretching exercise in which the participant position was more stable and that could have induced a greater activation of the upper limb muscles. Briefly, the participant was on one knee with the contralateral foot resting on the ground. From this position, the supporting knee was flexed, and the ipsilateral hip was extended. The right arm actively maintained the elongation. The forearm of the left limb was placed on a table to increase the stability of the position during the measurement and to allow the positioning of the finger photoplethysmography device without any constriction.

During SLKE and PST, the right arm was involved to stabilize the position of the participant on the ergometer in SLKE and to keep the elongation of the muscle involved in the stretching exercise in the PST, whereas the left arm was located on a support to allow the positioning of the finger photoplethysmography device without any constriction. A certain influence on the brachial artery vascular response because of the activation of the muscles of the upper limbs could, therefore, have occurred.

### Training

Both SLKE and PST were performed on both limbs and had a similar duration of 100 min · wk^−1^ for a total duration of 800 min (400 min each limb). To avoid overloading at knee extensors and knee joint level, SLKE was performed three times per week, with a single session duration of 33 min and 20 s. was performed five times per week, with a single session duration of 20 min. To enhance compliance, training sessions were held at different times of the day (morning and afternoon) at the university sports center gym. Sessions were supervised by expert operators who checked attendance, correct exercise execution, and intensity. Participants not attending at least 80% of classes were excluded from the study, and a new participant was recruited to substitute the dropout. The CTRL group received no training.

#### Passive static stretching training

**FIGURE 3:**
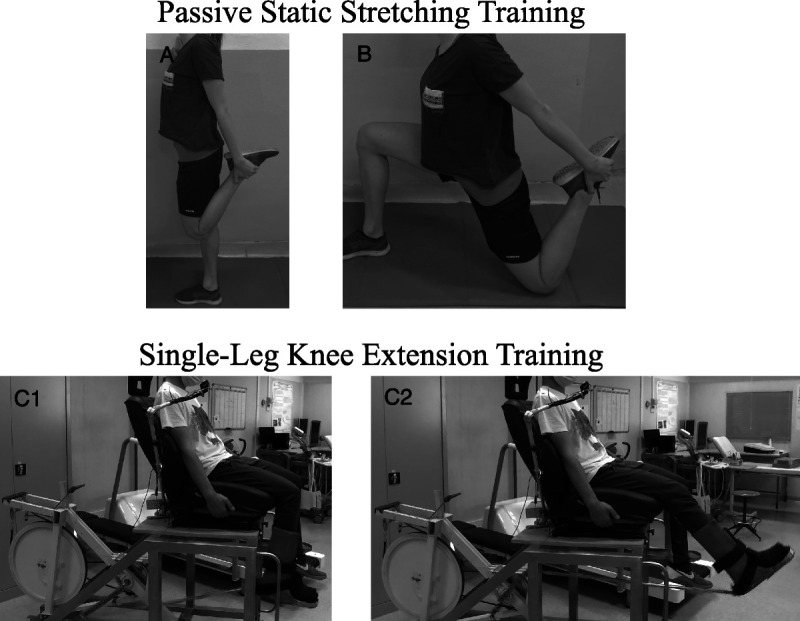
Panels A and B: Photographs showing the passive stretching exercises. Each exercise includes a set of five stretches of 45 s with 15 s of rest in between. Panel A, hip extension + knee flexion in orthostatism, Panel B, hip extension + knee flexion position. Panels C1 and C2. Photographic representation of participant’s positioning on the knee extension ergometer.

included 8 wk of PS training, five sessions per week (40 sessions in total). Each PST session lasted 20 min and included two exercises for the knee extensor muscles (Figs. 3A and B give an example of the PST exercises): 45-s elongation and 15-s recovery in the resting position; the set was repeated five times (24). The muscle elongation was maintained by the participant at the 80% of the point of discomfort (24).

#### SLKE muscles training

Participants underwent an 8-wk SLKE (three sessions per week, 24 sessions in total) involving both legs, one at a time. Single- was performed on the same ergometer used for W˙_max_ assessment, which allows participants to train only leg extensor muscles of a single limb (Fig. 3, panels C1 and C2). The leg starting order was balance among training sessions. Workloads ranged from 70% to 95% W˙_max_, with an average session duration of 16 min and 40 s for each leg (see Table, Supplemental Digital Content 1, Characteristics of each training session, http://links.lww.com/MSS/C446). The session’s workload was readjusted every 2 wk reassessing a new W˙_max_.

### Statistical Analysis

Statistical analyses were performed using a statistical software package (IBM SPSS Statistics v. 26, Armonk, NY). Based on previous research (18,24) for this study we used changes in *Q*˙ as main outcomes, a two-way ANOVA (within-group factor: time; between-groups factor: intervention) in the statistical approach, with an α level of 0.05 and a required power (1-β) of 0.80; the desired sample size computed using statistical software (G-Power 3.1, Dusseldorf, Germany) resulted in 36 participants.

The Shapiro–Wilk test was used to check the normal distribution of the sampling. A three-way ANOVA for repeated measures (RM, within-group factor: time, six levels [baseline + 5 phases], between-groups factor: condition, two levels (with or without active arm); phase (elongation or relaxation]) was applied to check for differences in *Y*˙ and MAP during the elongation and the relaxation phases occurring in an acute passive stretching bout. A two-way mixed-model ANOVA (within-group factor phase: six levels [baseline +5 phases]; between-groups factor: group, four levels [single-leg knee extension exercise without active arm, exercise with active arm, passive stretching without active arm, and passive stretching with active arm]) was applied to check for differences in *Y*˙ and MAP during the 5 min of exercises the acute passive stretching bout. To calculate the difference in changes between the groups, ANCOVA was performed, entering the baseline (PRE) values as a covariate.

A two-way mixed-model ANOVA (within-group factor: time, two levels [PRE, POST]; between-groups factor: group, three levels [SLKE, PST, and CTRL]) was used to assess the significant effects of training. To calculate the difference in changes between the groups, ANCOVA was performed, entering the baseline (PRE) values as a covariate. Multiple comparisons were performed applying Bonferroni’s correction. The ANOVA effect size was evaluated with partial eta squared (*η*_p_*^2^*) and classified as follows: <0.06: *small*; if, 0.06–0.14: *medium*; and >0.14: *large* (34). The Hedge’s *g* effect size with CI_95%_ was also calculated (https://www.cem.org/effect-size-calculator) and interpreted as follows: 0.00–0.19, trivial; 0.20–0.59, small; 0.60–1.19, moderate; 1.20–1.99, large; ≥2.00, very large (35). Data are presented in the mean (SD). Statistical significance was set with *P* < 0.05.

## RESULTS

### Participants’ compliance

Thirty-six low-active participants (18 women and 18 men; age, 22 yr [1 yr]; body mass, 68 kg [4 kg]; stature, 1.67 m [0.10 m], mean [standard deviation {SD}]) were enrolled and randomly assigned to one of three groups, balancing the sample for sex: SLKE (*n* = 12 [6 females/6 males]; age, 22 yr [2 yr]; body mass, 69 kg [3 kg]; stature, 1.68 m [0.10 m]); PST (*n* = 12 [6F/6M]; age, 23 yr [1 yr]; body mass, 68 kg [4 kg]; stature, 1.70 m [0.10 m]), and control group (no training, CTRL: *n* = 12 [6F/6M]; age, 22 yr [3 yr]; body mass, 69 kg [2 kg]; stature, 1.69 m [0.09 m]). Participants’ attendance was about 93% (268 of 288 training sessions) and 89% (427 of 480 training sessions) for SLKE and PST, respectively. Two participants for SLKE and one for PST dropped out throughout the study because of injury (unrelated to the training protocols) and lack of time. They were immediately replaced to maintain the required sample size.

### ROM, MVC, W_max_, muscles thickness, and cardiopulmonary parameters

Table 1 provided the PRE–POST changes in ROM, MVC, W˙_max_, muscle thickness, and in rest and peak values of *Q*˙_T_, q, HR, MAP, and V˙O_2_. ANOVA disclosed a significant time–group interaction in ROM, MVC, W˙_max_, muscle thickness, and V˙O_2_p. Range of motion increased only in PST in POST by 6% (3%) (*g*, 2.18 [1.17–3.20], *P* < 0.001). Conversely, MVC, W˙_max_, muscle thickness and V˙O_2_p increased only in SLKE by 23% (2%) (*g*, 1.67 [0.74–2.60]; *P* < 0.001), 24% (22%) (*g*, 0.84 [0.00–1.67]; *P* < 0.001), about 6% (4%) (*g*, from 0.45 to 0.84; *P,* from 0.001 to <0.001), and 15% (13%) (*g*, 0.85 [0.01–1.69]; *P* < 0.001), respectively. Mean arterial pressure decreased in SLKE and in SPT by −12% (4%) (*g*, −2.09 (−3.09–1.10); *P* < 0.001) and −7% (49%) (*g*, −0.99 [−1.84 to −0.14]; *P* < 0.001). No significant changes occurred in CTRL. ANCOVA disclosed significant differences in the increase for ROM in SPT compared with SLKE and CTRL (*g*, 1.28 and 0.97; *P* < 0.001, respectively) and for MVC (*g*, 1.69 and 1.68; *P* < 0.001), W˙_max_ (*g*, 4.99 and 4.98; *P* < 0.001), muscle thickness (*g*, from 0.42 to 1.52; *P,* from 0.004 to <0.001), and V˙O_2_p (*g*, 0.76 and 0.77; *P* = 0.001) in SLKE compared with SPT and CTRL. Mean arterial pressure decreased more after SLKE than PST and CTRL.

**TABLE 1:** Changes in knee joint ROM, MVC, and thickness of the knee extensor muscles, W˙max, and central hemodynamic parameters and POST SLKE (*n* = 12), PST (*n* = 12), and CTRL (*n* = 12).

Variable	Group	PRE m (SD)	POST Δ (CI_95%_)	ANOVA Time–Group Interaction
ROM (°)	SLKE	137 (4)	−0.2 (−1.5 to 1.9)	*F*, 23.24; *P* < 0.001; *η*_p_*^2^*, 0.592
	PST	135 (4)	−7.5 (−9.3 to −5.7)*^,^**^,^***	
	CTRL	137 (4)	−0.9 (−1.9 to 1.7)	
MVC (N)	SLKE	478 (57)	107 (102–112)*^,^***^,^****	*F*, 39.17; *P* < 0.001; *η*_p_*^2^*, 0.976
	PST	474 (56)	−1.2 (−6.2–3.9)	
	CTRL	472 (58)	−0.6 (−5.6–4.5)	
W˙max (W)	SLKE	52 (12)	11 (8–14)*^,^***^,^****	*F*, 20.52; *P* < 0.001; *η*_p_*^2^*, 0.562
	PST	50 (11)	−0.2 (−3.0 to 2.6)	
	CTRL	53 (11)	−0.4 (−3.2 to 2.5)	
Vastus lateralis thickness (mm)	SLKE	24 (2)	2.1 (1.0–3.2)*^,^***^,^****	*F*, 2.77; *P* < 0.070; *η*_p_*^2^*, 0.148
	PST	22 (4)	0.5 (−1.7 to 0.5)	
	CTRL	24 (4)	0.6 (−0.5 to 1.7)	
Vastus intermedius thickness (mm)	SLKE	18 (2)	1.0 (0.5–1.6)*^,^***^,^****	*F*, 4.70; *P* < 0.019; *η*_p_*^2^*, 0.218
	PST	17 (2)	0.2 (−0.4 to 0.4)	
	CTRL	16 (3)	0.1 (−0.4 to 0.6)	
Rectus femoris thickness (mm)	SLKE	20 (2)	1.0 (0.3–1.8)*^,^***^,^****	*F*, 7.17; *P* < 0.003; *η*_p_*^2^*, 0.309
	PST	20 (2)	0.8 (−0.9 to 1.5)	
	CTRL	19 (2)	−0.3 (−1.0 to 0.4)	
Q˙_T_ rest (L·min^−1^)	SLKE	5.6 (1.0)	−0.1 (−0.5 to 0.2)	*F*, 0.38; *P* = 0.685; *η*_p_*^2^*, 0.02
	PST	5.5 (1.0)	0.03 (−0.3 to 0.4)	
	CTRL	5.7 (1.0)	0.05 (−0.4–0.4)	
Q˙_T_ peak (L·min^−1^)	SLKE	16.4 (3.8)	−0.2 (−1.1 to 0.8)	*F*, 0.05; *P* = 0.950; *η*_p_*^2^*, 0.003
	PST	16.6 (3.9)	0.3 (−0.9 to 0.9)	
	CTRL	16.3 (3.7)	0.3 (−0.9 to 1.0)	
q rest (mL)	SLKE	79 (13)	−2.9 (−6.3 to 0.6)	*F*, 0.89; *P* = 0.419; *η*_p_*^2^*, 0.053
	PST	80 (11)	0.3 (−3.2 to 4.3)	
	CTRL	78 (10)	−0.8 (−4.3 to 2.6)	
q peak (mL)	SLKE	112 (17)	−0.3 (−2.8 to 2.1)	*F*, 0.06; *P* = 0.938; *η*_p_*^2^*, 0.004
	PST	111 (16)	−0.4 (−2.9 to 2.0)	
	CTRL	113 (15)	0.1 (−2.3 to 2.6)	
HR rest (bpm)	SLKE	71 (8)	−0.4 (−3.1 to 2.4)	*F*, 0.12; *P* = 0.884; *η*_p_*^2^*, 0.008
	PST	70 (8)	−0.1 (−2.8 to 2.7)	
	CTRL	72 (9)	−1.0 (−3.7 to 1.7)	
HR peak (bpm)	SLKE	146 (19)	−0.5 (−7.4 to 6.5)	*F*, 0.06; *P* = 0.940; *η*_p_*^2^*, 0.004
	PST	145 (17)	1.2 (−5.7 to 8.2)	
	CTRL	144 (15)	0.2 (−6.8 to 7.1)	
MAP (mm Hg)	SLKE	88 (5)	−10.2 (−13 to −7)*^,^***^,^****	*F*, 9.52; *P* = 0.001; *η*_p_*^2^*, 0.373
	PST	87 (6)	−6.3 (−9 to −4)*^,^**^,^***	
	CTRL	88 (7)	−1.8 (−4 to 1)	
V˙O_2_ rest (mL·kg^−1^·min^−1^)	SLKE	4.07 (0.7)	0.03 (−0.3 to 0.3)	*F*, 0.194; *P* = 0.824; *η*_p_*^2^*, 0.01
	PST	4.16 (0.6)	−0.06 (−0.4 to 0.2)	
	CTRL	4.05 (0.6)	−0.09 (−0.4 to 0.3)	
V˙O_2_ peak (mL·kg^−1^·min^−1^)	SLKE	22.5 (3.4)	3.0 (2.0–4.0)*^,^***^,^****	*F, 13.20; P < 0.001; η*_p_ *^2^, 0.452*
	PST	21.6 (3.3)	0.1 (−1 to 1)	
	CTRL	22.8 (3.2)	−0.1 (−1 to 1)	

Two-way mixed model ANOVA (time–groups); ANCOVA detected differences in changes between the groups. *P < 0.05 vs baseline (PRE), **P < 0.05 vs SLKE, ***P < 0.05 vs CTRL, ****P < 0.05 vs PST.

### sPLM

**FIGURE 4:**
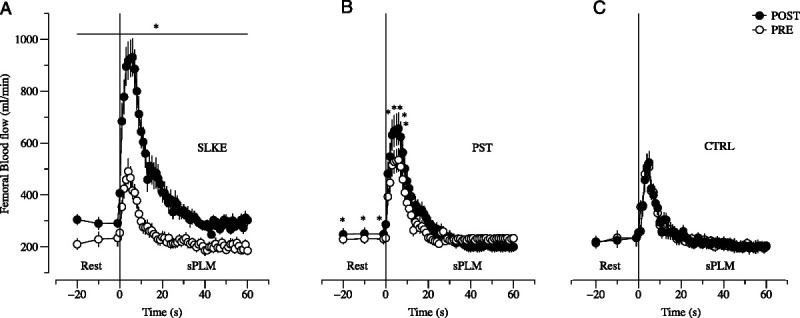
Changes in femoral blood flow induced by single passive limb movement test PRE (*open circle*) and POST (*closed circle*) in training (panel A, *n* = 12), passive static stretching training (PST, panel B, *n* = 12), and control group (CTRL, panel C, *n* = 12). Two-way mixed model ANOVA (time–groups); ANCOVA detected differences in changes between the groups. *P < 0.05 vs baseline (PRE).

Table 2 and Figure 4 demonstrated the PRE–POST difference in the sPLM variables. ANOVA disclosed significant time–group interactions in all the variables. Rest, peak, and Δ*Q*˙_fem_ increased in SLKE and PST by 35% (13%) and 7% (5%) (SLKE *g*, 0.91 [0.07–1.75]; *P* = 0.01; PST *g*, 0.51 [0.30–1.33]; *P* = 0.02), by 47% (4%) and 14% (2%) (SLKE *g*, 3.71 [2.39–5.03]; *P* < 0.001; PST *g*, 2.26 [1.24–3.29]; *P* < 0.001), and by 54% (7%) and 20% (2%) (SLKE *g*, 2.72 [1.61–3.83]; *P* < 0.001; PST *g*, 2.43 [1.38–3.49]; *P* < 0.001). Area under the curve increased in POST in SLKE by 60% (11%) (*g*, 2.06 [1.07–3.05]; *P* < 0.001) and in PST by 11% (3%) (*g*, 0.76 (0.07–1.59; *P* = 0.003). No significant changes occurred in CTRL. ANCOVA evidenced increments in all sPLM variables larger in SLKE than in PST and CTRL (*g*, from 0.24 to 2.95; *P,* from 0.03 to <0.001).

**TABLE 2:** Changes in femoral artery blood flow during the sPLM test POST SLKE (*n* = 12), PST (*n* = 12), and CTRL (*n* = 12).

sPLM Variables	Group	PRE m (SD)	POST Δ (CI_95%_)	ANOVA Time–Group Interaction
*Q*˙_fem_ rest (mL·min^−1^)	SLKE	200 (20)	68 (27–107)*^,^**^,^***	*F*, 4,16; *P* = 0.04; *η*_p_*^2^*, 0.185
	PST	234 (12)	11 (2–29)*^,^***^,^****	
	CTRL	230 (12)	2 (−35 to 39)	
*Q*˙_fem_ peak (mL·min^−1^)	SLKE	570 (32)	270 (234–306)*^,^**^,^***	*F*, 32.00; *P* < 0.001; *η*_p_*^2^*, 0.790
	PST	540 (29)	73 (40–107)*^,^***^,^****	
	CTRL	537 (25)	−4 (−38 to 30)	
Δ*Q*˙_fem_ (mL·min^−1^)	SLKE	370 (27)	186 (136–237)*^,^**^,^***	*F*, 14.09; *P* < 0.001; *η*_p_*^2^*, 0.468
	PST	306 (16)	69 (32–107)*^,^***^,^****	
	CTRL	307 (14)	3 (−34 to 40)	
AUC (mL)	SLKE	56 (7)	33 (26–40)*^,^**^,^***	*F*, 26.18; *P <* 0.001; *η*_p_*^2^*, 0.621
	PST	52 (6)	5 (0.9–12)*^,^***^,^****	
	CTRL	53 (6)	0.06 (−7 to 7)	

*Q*˙_fem_ rest: femoral artery blood flow at rest, *Q*˙_fem_ peak: peak femoral artery blood flow, Δ*Q*˙_fem_: difference between peak and rest femoral artery blood flow. Two-way mixed model ANOVA (time–groups); ANCOVA detected differences in changes between the groups. *P < 0.05 vs baseline (PRE); **P < 0.05 vs PST, ***P < 0.05 vs CTRL. ****P < 0.05 vs SLKE.

### FMD

**FIGURE 5:**
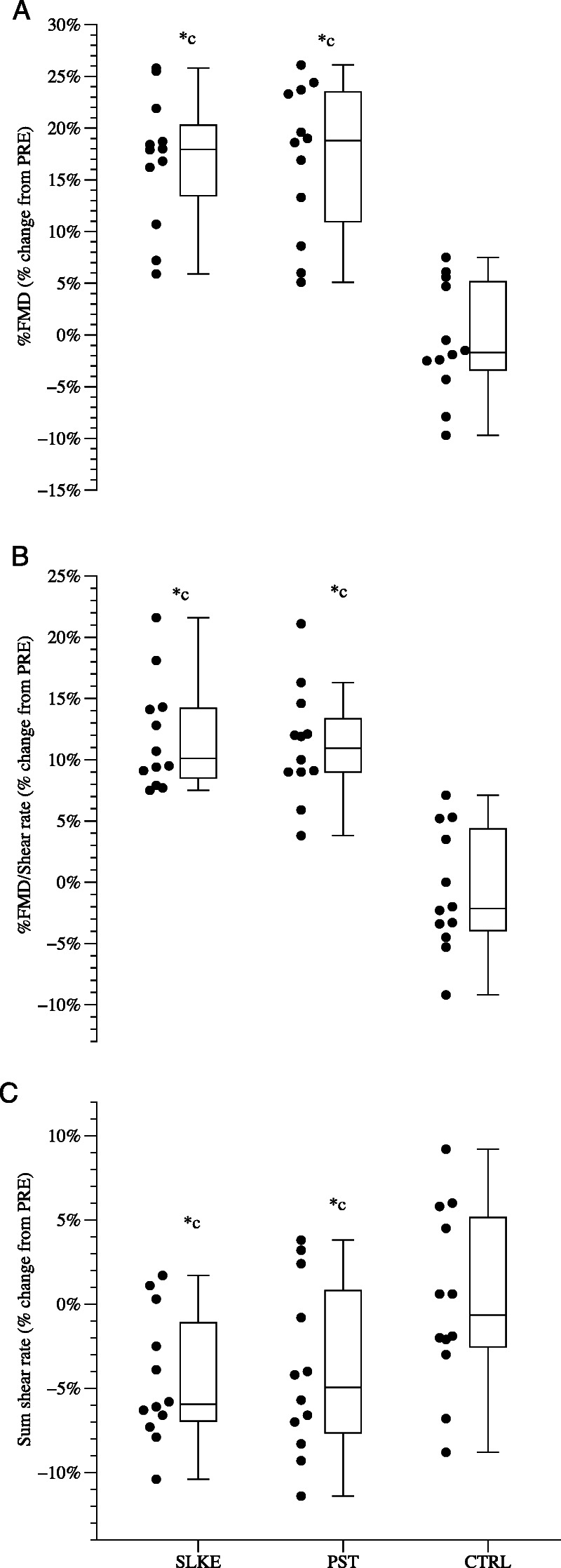
Individual percentage changes in %FMD over *Y*˙, %FMD: flow-mediated dilation in percentage, and sum of *Y*˙ after training (*n* = 12), passive static stretching training (PST, *n* = 12), and control group (CTRL, *n* = 12). Box and whisker plot reports the median values together with the standard deviation, standard error, and the minimum-maximum range. Two-way mixed model ANOVA (time–groups); ANCOVA detected differences in changes between the groups. *P < 0.05 vs baseline (Pre), ^c^*P* < 0.05 vs CTRL.

Table 3 and Figure 5 illustrate the PRE–POST differences in the FMD variables. Except for rest diameter and *Y*˙, ANOVA found significant time–group interactions in all the other variables. Peak diameter increased in POST in SLKE and PST by 6% (2%) (*g*, 0.54 [0.26–1.06]; *P* = 0.007) and 4% (1%) (*g*, 0.54 [0.27–1.36]; *P* < 0.001), respectively. In POST %FMD increased by 12% (2%) (*g*, 0.68 [0.14–1.51]; *P* < 0.001) and 11% (1%) (*g*, 0.83 [0.04–1.66]; *P* < 0.001) in SLKE and PST. FMD/*Y*˙ increased in POST by 18% (2%) (*g*, 0.66 [0.16–1.49]; *P* < 0.001) and 17% (2%) (*g*, 0.67 [0.15–1.49]; *P* < 0.001) in SLKE and PST. In POST, AUC increased by 14% (7%) (*g*, 0.60 [0.22–1.42]; *P* < 0.001) and 10% (3%) (*g*, 0.66 [0.16–1.48]; *P* < 0.001) in SLKE and PST, respectively. No changes occurred in CTRL in any variables. Remarkably, ANCOVA disclosed similar PRE–POST increments in SLKE and PST in all the FMD variables, which were significantly larger compared with CTRL (*g*, from 0.64 to 1.84; *P,* from 0.002 to <0.001).

**TABLE 3:** Changes in brachial artery vascular responsiveness variables during the FMD POST SLKE (*n* = 12), PST (*n* = 12), and CTRL (*n* = 12).

FMD Variables	Group	PRE, m (SD)	POST, Δ (CI_95%_)	ANOVA, Time–Group Interaction
Rest diameter (cm)	SLKE	0.30 (0.06)	0.01 (0.00–0.02)	*F*, 0.756; *P* = 0.417; *η*_p_*^2^*, 0.05
	PST	0.31 (0.02)	0.01 (−0.01 to 0.01)	
	CTRL	0.32 (0.02)	0.00 (−0.01 to 0.01)	
Peak diameter (cm)	SLKE	0.35 (0.07)	0.017 (0.016–0.018)*^,^**	*F*, 11.85; *P <* 0.001; *η*_p_*^2^*, 0.426
	PST	0.36 (0.03)	0.014 (0.013–0.015)*^,^**	
	CTRL	0.37 (0.03)	−0.002 (−0.007 to 0.005)	
%FMD (%)	SLKE	15.9 (2.6)	1.92 (1.91–1.94)*^,^**	*F*, 19.37; *P <* 0.001; *η*_p_*^2^*: 0.547
	PST	16.6 (2.0)	1.83 (1.81–1.84)*^,^**	
	CTRL	17.0 (3.4)	−0.21 (−0.23 to 0.20)	
$\overset{\cdot }{Y}$ (s^−1^, ×1000)	SLKE	155 (21)	−6.7 (−6.8 to −6.6)	*F*, 1.12; *P* = 0.43; *η*_p_*^2^*, 0.058
	PST	146 (18)	−7.6 (−7.7 to −7.4)	
	CTRL	151 (18)	−5.5 (−6.5 to 4.9)	
	SLKE	0.011 (0.002)	0.002 (0.002–0.003)*^,^**	*F*, 21.11; *P* < 0.001; *η*_p_*^2^*, 0.660
FMD/*Y*˙ (%·s^−1^)	PST	0.012 (0.003)	0.002 (0.002–0.003)*^,^**	
	CTRL	0.012 (0.003)	0.000 (−0.001 to 0.001)	
	SLKE	37.5 (7.2)	5.3 (4.0–6.6)*^,^**	*F*, 18.54; *P* < 0.001; *η*_p_*^2^*, 0.537
AUC (mL)	PST	38.3 (4.9)	3.7 (2.4–5.0)*^,^**	
	CTRL	40.2 (6.2)	−0.1 (−1.4 to 1.2)	

Two-way mixed model ANOVA (time–groups); ANCOVA detected differences in changes between the groups. *P < 0.05 vs baseline (PRE), **P < 0.05 vs CTRL.

### Acute hemodynamic measurements

Table 4 panel A demonstrated the acute changes in the mean *Y*˙ at brachial artery level and the MAP during the 5-min and the single bout of passive stretching. Two-way ANOVA reported no time–condition interaction for the mean $\overset{\cdot }{Y}$ and MAP during the and the passive stretching bout. Main effects for time were retrieved for the mean *Y*˙ and MAP during the and the passive stretching bout. ANCOVA disclosed significant differences in the increases in MAP between the and the passive stretching (*g,* from 1.13 to 4.43; *P,* from 0.02 to <0.001). On the contrary, no differences were shown for changes in *Y*˙ between the different conditions.

**TABLE 4:** Acute shear rate (*Y*·) and mean arterial pressure (MAP) changes during the single knee extension muscle exercise and knee extensor muscles stretching bout performed without and with the active arm.

										
A		Baseline	1st Min	2nd Min	3rd Min	4th Min	5th Min	Time–Condition Interaction	Time	ANCOVA
SLKE without arm (*n* = 12)	$\overset{\cdot }{Y}$ (s)	17 (1)	19 (3)	20 (3)*	22 (3)*	23 (3)*	23 (3)*	*F*, 1.599	*F*, 3.826	*F*, 1.928
SLKE with active arm (*n* = 12)		16 (3)	18 (3)*	19 (3)*	20 (3)*	22 (3)*	22 (3)*	*P* = 0.090	*P* = 0.003	*P* = 0.123
PS without arm (*n* = 12)		18 (3)	21 (4)*	20 (4)*	21 (5)	20 (3)*	20 (3)	*η*_p_*^2^*, 0.067	*η*_p_*^2^*, 0.110	*η*_p_*^2^*, 0.063
PS with active arm (*n* = 12)		19 (3)	22 (5)*	23 (4)*	22 (4)*	21 (4)	22 (5)
SLKE without arm (*n* = 12)	MAP (mm Hg)	88 (8)	102 (11)*^,^**	109 (11)*^,^**	117 (6)*^,^**	120 (8)*^,^**	121 (8)*^,^**	*F*, 27.661	*F*, 12.364	*F*, 8.509
SLKE with active arm (*n* = 12)		88 (5)	105 (4)*^,^**	106 (4)*^,^**	112 (5)*^,^**	118 (5)*^,^**	119 (5)*^,^**	*P* < 0.001	*P* < 0.001	*P* < 0.001
PS with active arm (*n* = 12)		89 (8)	88 (6)	89 (6)	89 (8)	89 (6)	89 (6)	*η*_p_*^2^*, 0.755	*η*_p_*^2^*, 0.314	*η*_p_*^2^*, 0.240
PS with active arm (*n* = 12)		89 (8)	89 (5)	89 (5)	89 (7)	89 (5)	89 (6)

B		Passive Stretching Bout						Time–Condition–Phase Interaction	Time–Condition Interaction	Time–Phase Interaction
	Elongation	Baseline	1st Min	2nd Min	3rd Min	4th Min	5th Min			
PS without active arm (*n* = 12)	$\overset{\cdot }{Y}$ (s^−1^)	18 (3)	17 (3)***	16 (2)***^,^****	16 (4)***^,^****	16 (3)***^,^****	16 (3)***^,^****	*F*, 1.592	*F*, 0.975	*F*, 11.789
PS with active arm (*n* = 12)		19 (3)	19 (6)***	18 (5)***	18 (4)***^,^****	17 (4)***^,^****	19 (5)***	*P* = 0.165	*P* = 0.435	*P* < 0.001
	Relaxation							*η*_p_*^2^*, 0.042	*η*_p_*^2^*, 0.026	*η*_p_*^2^*, 0.247
PS without active arm (*n* = 12)	$\overset{\cdot }{Y}$ (s)	18 (3)	24 (5)****	25 (6)****	26 (7)***^,^****	24 (3)****	23 (4)***^,^****
PS with active arm (*n* = 12)		19 (3)	26 (6)****	28 (5)****	25 (5)****	23 (6)****	25 (7)****
PS without active arm (*n* = 12)	MAP (mm Hg)	89 (8)	91 (5)***	93 (5)***^,^****	92 (8)***^,^****	92 (6)***^,^****	91 (5)***	*F*, 0.479	*F*, 0.309	*F*, 15.881
PS with active arm (*n* = 12)		89 (8)	91 (4)***	94 (4)***^,^****	92 (7)***^,^****	94 (4)***^,^****	93 (5)***^,^****	*P* = 0.792	*P* = 0.907	*P* < 0.001
	Relaxation							*η*_p_*^2^*, 0.017	*η*_p_*^2^*, 0.011	*η*_p_*^2^*, 0.362
PS without active arm (*n* = 12)	MAP (mm Hg)	89 (8)	86 (6)	85 (7)****	86 (8)****	86 (7)****	86 (7)****
PS with active arm (*n* = 12)		89 (8)	86 (5)	85 (7)****	85 (8)****	85 (7)****	85 (8)****

Panel A: Changes in the mean *Y*˙ in the brachial artery (upper table) and MAP (lower table) detected at baseline and during the 5-min acute SLKE muscle exercise performed at 70% of the maximum work rate and during the acute stretching bout administered to the knee extensor muscles performed without and with the active arm. ANCOVA detected possible intercondition differences in $\overset{\cdot }{Y}$ and in MAP.

Panel B: Changes in the mean *Y*˙ in the brachial artery and MAP detected at baseline and during the elongation and relaxation phases of the acute stretching bout administered to the knee extensor muscles (*n* = 12) with and without the active arm. Three-way repeated-measures ANOVA (time–conditions–phase).

*P < 0.05 vs baseline, **P < 0.05 SLKE (with and without active arm) vs passive stretching (with and without active arm).

***P < 0.05 elongation vs relaxation, ****P < 0.05 vs baseline.

During the 5-min, *Y*˙ without active arm increased compared with the baseline from the second to the fifth minutes by on average 28% (20%) (*g,* from 0.64 to 2.96; *P,* from 0.017 to 0.008), and with active arm from the first to the fifth minutes by on average 26% (11%) (*g,* from 0.59 to 1.15; *P,* from 0.027 to 0.005). During the 5-min passive stretching bout, *Y*˙ without active arm increased compared with the baseline in the first, third, and, fourth minutes by, on average, 13% (3%) (*g,* from 0.35 to 0.82; *P,* from 0.008 to <0.001), and with active arm from the second to the third minutes by on average 16% (4%) (*g,* from 0.32 to 0.90; *P,* from 0.016 to 0.019).

MAP increased only during the from the first to the fifth minutes in both conditions, by, on average, 30% (16%) (*g,* from 1.41 to 4.06; *P,* from 0.039 to 0.001), and 23% (11%) (*g,* from 4.18 to 4.56; *P,* from 0.002 to <0.001), without and with the active arm, respectively.

Table 4 panel B reported the changes in *Y*˙ and MAP during the and the elongation and relaxation phase of passive stretching in the two conditions. Three-way ANOVA reported no time–condition–phase and no time–condition interaction, but a significant time–phase interaction. No between-conditions differences were found. During the elongation phase, *Y*˙ decreased compared with the baseline from the second to the fifth elongation by on average −14% (8%) (*g,* from −1.03 to −0.79; *P,* from 0.017 to 0.001) without active arm and during the fourth and fifth elongations with active arm (−8% [7%]; *g* = −0.47 and −0.65; *P,* from 0.03 to 0.01). Mean arterial pressure without active arm increased from the second to the fourth elongation by −4 (2)% (*g*, from −0.61 to −0.31; *P,* from 0.02 to 0.007), and MAP with active arm from the second to the fifth elongation by −5% (2%) (*g,* from −0.80 to −0.45; *P,* from 0.04 to 0.02).

During the relaxation phase, the mean *Y*˙ without active arm increased during the five maneuvers by 30% (12%) (*g,* from 1.26 to 1.60; *P,* from 0.017 to <0.001), and with active arm by 33% (11%) (*g,* from 0.87 to 2.04; *P,* from 0.04 to <0.001). Mean arterial pressure without active arm decreased from the second to the fifth maneuver by −4% (2%) (*g,* from −0.80 to −0.45; *P,* from 0.04 to 0.02), and with active arm by −5% (2%) (*g,* from −0.52 to −0.34; *P,* from 0.04 to 0.01).

## DISCUSSION

This study evaluated the local and systemic vascular responsiveness adjustments induced by an 8-wk SLKE or PST intervention. In line with our hypothesis, SLKE induced greater improvements in local vascular responsiveness than PST. Surprisingly, SLKE and PST led to similar vascular responsiveness improvements at the systemic level. Although, locally, the action of multiple concomitant mechanisms triggered a greater increase in vascular responsiveness after SLKE, at the systemic level, the similar changes in *Y*˙ produced by the two training regimens were likely the reason for the equal increases in vascular responsiveness.

### Preliminary consideration

Similar to previous studies (18,24,36), both SLKE and PST have shown their effectiveness. An improvement in peak V˙O_2_, W˙_max_, and MVC of the knee extensor muscles was indeed already reported after SLKE of equal duration (but lower intensity) in a group of moderately active young people (18). These adaptations occurred with no changes in heart hemodynamics because of the minimum taxing of this small muscle mass exercise paradigm on central factors. These findings are also in line with previous reports in health and disease (17,37,38), indicating that this specific training paradigm can induce improvements in peripheral convective and diffusive oxygen transport aspects without detectable changes in central hemodynamics.

An increase in ROM without any change in knee extensor muscle MVC occurred after the 8-wk PST. These findings are in line with a previous investigation, in which an increase in ROM coupled to a preserved MVC of the plantar flexor muscles was reported after 12-wk PST (36).

Under a technical point of view, although we followed the recommended technique and analysis to determine %FMD, the %FMD values are quite a bit higher than typically expected (39). The involvement of young, healthy, and sedentary participants may possibly explain the higher % FMD values reported here.

### SLKE and PST effects on local vascular responsiveness

The sPLM response increased after both SLKE and PST but with ameliorations that were from fourfold to fivefold larger in the former than in the latter training modality. Previous investigations indicated that sPLM response can provide important insights into vascular responsiveness, being more representative of a microvascular assessment and closely related to local vasodilatory factors (30,40). The most representative sPLM variables involve the following: (i) the peak flow; (ii) the change from baseline to peak flow (∆ peak flow); and (iii) AUC (30,41). As previously reported, all these parameters are related to NO bioavailability (41). Considering vascular tube length and blood viscosity relatively constant, and applying Poiseuille’s law, sPLM-induced hyperemia may be driven by two main factors: an increase in perfusion pressure and peripheral vasodilation (i.e., drop in the peripheral resistance). Our data reveal an increase in the *Q*˙_fem_ at rest after both SLKE and PST, together with a lower MAP. These findings may be ascribed to a reduction in total peripheral vascular resistance that led to an increased muscle perfusion. Indeed, sPLM causes the mechanical deformation of the vessels determining vasoactive-substances release, among which NO (42,43), which, in turn, results in the dilatation of the vascular bed (31). Therefore, considering the nature of this hyperemic response, we can ascribe the sPLM-induced hyperemia, at least in part, related to an enhanced NO bioavailability (30,44). The oscillations in *Q*˙ during the two interventions and, in turn, in *Y*˙, could have resulted in an important stimulus to the endothelial cell membrane that triggered a cascade of signaling events favoring NO bioavailability (18,24,27).

As previously suggested, the amplitude of vascular responsiveness improvement seems to be *Y*˙ magnitude-dependent (9). Our results implied that the *Y*˙ in the femoral artery produced during SLKE was higher compared with PST, thus producing in the former a larger improvement in local vascular responsiveness. Greater *Y*˙during SLKE would be the effect of greater metabolic demand by the muscles involved with exercise. A greater increase in local vascular responsiveness after SLKE compared with PST could be further explained by the increase in muscle thickness that was induced only after SLKE. An increased muscle volume leads to a greater microcirculation, a phenomenon that has an important impact on the response of the sPLM (8,45). Instead, regarding the improvement of local vascular responsiveness after PST, an additional mechanism that should be considered is the possible change in vessel tortuosity. Indeed, the enhancement of the microvasculature, coupled with a lower MAP, could be explained by a reduction in total peripheral vascular resistance. Vessel tortuosity is linked to this resistance in large and small blood vessels and to *Q*˙ distribution (46). Vessel tortuosity describes how twisted the capillaries are and how many turns and bends they have, on which basis a physiological index of the capillary extension reserve is defined (46,47). Changes in muscle sarcomere length reduce significantly the capillary extension reserve (i.e., tortuosity) (46,47). Extensive muscle lengthening (as in PST) results in repeated cycles of vessel elongation and compression, leading to a reduction in vessel resistance and capillary diameter, as well as in *Q*˙ and O_2_ supply (47). The acute vessel distortion is an important stimulus for long-term vascular adaptations induced by PST. While our study did not supply direct evidence for changes in vessel tortuosity, its reduction following PST cannot be ruled out. Lastly, the continuous oscillations of *Q*˙, and therefore of *Y*˙, induced by active and passive exercise have been advocated as possible mechanisms for the increase in the levels of vascular endothelial growth factor protein, in endothelial cell proliferation rate, and in eNOS messenger RNA levels, all aspects that can improve vascular responsiveness (48,49). The lack of metabolic activation of the muscles during stretching compared with active exercise could justify the differences seen in local vascular responsiveness reported here. Although vascular endothelial growth factor protein levels, endothelial cell proliferation rate, and endothelial NOS messenger RNA levels before and after training were not evaluated in the present study, their possible contribution to local vascular responsiveness enhancement may play a role.

### SLKE and PST effects in systemic vascular responsiveness

Eight weeks of SLKE and PST led to an improvement also in brachial artery vascular responsiveness, an artery distal from the application point of the mechanical stimulus. The acute measurement of *Q*˙ has shown that significant changes in *Y*˙ at the level of the brachial artery occurred during both active and passive exercises, which were accompanied by a concomitant adaptation of the MAP. Although some activation of the upper-limb muscles occurred during the two training modalities (during SLKE to stabilize the participant in the ergometer and during PST to keep in elongation the stretched muscles), the acute changes in *Y*˙and MAP were similar between the passive condition (i.e., without active arm) and the condition with the intervention of the upper limb muscles (i.e., with active arm). This suggests that the contribution of the activation of the upper limb muscle to the improvement of the brachial artery vascular responsiveness could have been minimal, and therefore, the improvements in the brachial artery could be mainly because of the changes in $\overset{\cdot }{Y}$ induced by the two training modalities.

The continuous changes in $\overset{\cdot }{Y}$ during the two training methods may have contributed to an improvement in vascular responsiveness. Surprisingly, the changes in *Y*˙ were not different between the two interventions, suggesting a systemic response not proportional to the muscular effort required during active and passive exercise. An increase in vascular responsiveness after SLKE and PST is in line with previous reports, which observed an improvement in some central mechanisms of *Q*˙ control as a possible explanation for the systemic ameliorations indicated by the FMD increment in the brachial artery. It should be kept in mind that the FMD response is strongly correlated with the dilation capacity at the coronary level (3). Therefore, the improvement in systemic vascular responsiveness detected in the present study could also have important implications on cardiovascular risk.

The present study did not allow to evaluate whether the vascular responsiveness ameliorations retrieved at the brachial artery level could have been induced by circulating levels of vascular endothelial growth factor, likely triggered by the active and passive remote exercise training. Although further studies will be needed to evaluate this mechanism, its possible involvement in explaining systemic changes in vascular responsiveness cannot be ruled out. Contrary to the present work, a previous investigation did not reveal a systemic vascular responsiveness improvement following 8 wk of SLKE (18). A larger sample size included in the present investigation could possibly justify the differences that emerged between the two studies.

### Study Limitations

The present study comes with some limitations. First, although the effect of the upper limb muscles in increasing the $\overset{\cdot }{Y}$ in the brachial artery was minimal, in the long term, it might have improved the brachial artery responsiveness due to local instead of systemic mechanisms only. Second, the sPLM and FMD represent the function of two distinct types of vessels and are not representative of similar mechanisms, being sPLM response correlates to the resistance artery or microcirculatory function, whereas FMD represents the function of the conduit artery. However, they could provide evidence for an improved overall vascular responsiveness at the local (sPLM) and systemic (FMD) level. To have a clearer evaluation of the effect of training on the local and systemic blood flow control mechanisms, the last should involve conduit arteries or resistance arteries of both the upper and lower limbs. Third, direct assessment of muscle sympathetic nerve activity, NO bioavailability, and vascular endothelial growth factor protein levels would have confirmed possible training-induced modulation of sympathetic vessel tone and endothelial function. The lack of a direct assessment of these measurements did not allow us to establish a clear balance between central and local mechanisms underlying the positive vascular responsiveness changes. Fourth, the evaluation of local and systemic vascular responsiveness parameters after a follow-up period could have highlighted any differences between the two interventions in the persistence of their effects. Unfortunately, because of the COVID-19 pandemic situation and the limitations imposed on the laboratory’s attendance, it was not possible to plan a further evaluation related to the follow-up. Lastly, the systemic vascular effects reported in this study refer only to the comparison between PST and SLKE and do not give indications with respect to the systemic vascular adaptations obtainable with endurance training involving large muscle masses, such as cycling and running (which should induce larger systemic adaptations compared with PST).

## CONCLUSIONS

Despite involving similar muscle mass (knee extensor muscles), larger increases in local vascular responsiveness after active (SLKE) than after passive training (PST) occurred. Surprisingly, similar systemic vascular responsiveness improvements (similar increases in FMD parameters at the level of the brachial artery) were observed after both interventions. While at the local level the greater variations of *Q*˙ (and therefore of $\overset{\cdot }{Y}$) during SLKE may have led to a more evident vascular responsiveness improvement. On the contrary, FMD findings would suggest that the vascular responsiveness at the systemic level was not affected in a directly proportional way to the muscular demands. From a practical point of view, these results would indicate how also a passive method (PST) can trigger significant improvements in systemic vascular responsiveness. This would be particularly crucial given the importance of maintaining or even improving vascular responsiveness in all those individuals with limited mobility, to whom an active method would not be accessible.

## Supplementary Material
