# Supplementary material for: Long-Term Passive Leg Stretch Improves Systemic Vascular Responsiveness as Much as Single-Leg Exercise Training
Source: Med Sci Sports Exerc. 2021 Oct 25;54(3):475–88. doi: 10.1249/MSS.0000000000002811 (PMC10097495; doi:10.1249/MSS.0000000000002811)
Supplement: Supplementary file 1 [file msse-54-475-s001.docx]

**Long-Term Passive Leg Stretch Improves Systemic Vascular Responsiveness as much as Single-Leg Exercise Training**

Emiliano Cè^1,2,*^, Massimo Venturelli^3,4,*^, Angela Valentina Bisconti^1,4^, Stefano Longo^1^, Anna Pedrinolla^3^, Giuseppe Coratella^1^, Federico Schena^3^, Fabio Esposito^1,2^

^*^These authors contributed equally

^1^Department of Biomedical Sciences for Health, Università degli Studi di Milano, Milan, Italy; ^2^IRCCS Istituto Ortopedico Galeazzi, Milan, Italy; ^3^Department of Neuroscience, Biomedicine, and Movement Science, Section of Movement Science, University of Verona, Verona, Italy; ^4^Department of Internal Medicine section of Geriatrics, University of Utah, Salt Lake City, UT

**Short title:** Stretching improves vascular function

*Corresponding author:*

Prof. Fabio Esposito

Department of Biomedical Sciences for Health (SCIBIS)

University of Milan

Via Colombo 71

20133 Milan, Italy

E-Mail: [fabio.esposito@unimi.it](mailto:fabio.esposito@unimi.it)

Orcid ID: 0000-0003-0691-3153

The present study was funded by a dedicated grant (PSR Fondi Linea 2 -Tipologia A - 2016_CE) from the Department of Biomedical Sciences for Health, Università degli Studi di Milano. The authors have no conflict of interest. The results of the study are presented clearly, honestly, and without fabrication, falsification, or inappropriate data manipulation, and statement that results of the present study do not constitute endorsement by ACSM.

**ABSTRACT**

**Purpose**: The current study compared the local and systemic vascular responsiveness after small muscle mass endurance training or passive stretching training (PST). **Methods**: Thirty-six sex-matched healthy participants underwent 8-week single-leg knee extension (SLKE) (N=12) training or PST (N=12), or no intervention (control, N=12). Before and after the intervention, local and systemic vascular responsiveness was assessed by Doppler ultrasound at the femoral (local effect) and brachial artery (systemic effect) during single passive leg movement (sPLM) and brachial flow-mediated dilation (FMD) test, respectively. **Results**: After training, delta femoral blood flow (representing the local vascular responsiveness) increased after SLKE and PST by +54(7)% (effect size: 2.72, *P*<0.001) and +20(2)%, (effect size: 2.43, *P*<0.001), respectively, albeit with a greater extent in SLKE [post-SLKE *vs.* post-PST: +56(8)%, (effect size: 2.92, *P*<0.001)]. Interestingly, the %FMD (standing for the systemic effect) increased after SLKE and PST by +12(2)% (effect size: 0.68, *P*<0.001) and +11(1)% (effect size: 0.83, *P*<0.001), respectively, without any between-groups difference (*P*>0.05). No changes occurred in control. **Conclusion**: The present findings revealed that both active and passive training modalities induced similar improvements in the brachial artery dilatation capacity, while the former was more effective in improving femoral artery blood flow. Passive stretching could be used in people with limited mobility to improve vascular responsiveness both at the local and systemic level, and in this latter case has similar effects as small muscle mass endurance training. **Keywords:** flow-mediated dilation; shear rate; single-leg knee extension training; single passive limb movement; vascular conditioning; vascular plasticity.

**INTRODUCTION**

Vascular responsiveness is the ability of the arterial tree to dilate and constrict and represents an important marker of cardiovascular health and a predictor of cardiovascular diseases (1–4). Vascular responsiveness decreases with advancing age and is compromised in people with heart disease, metabolic alterations, or limited mobility (5–10), determining a further increase in the cardiovascular risk in these populations. Indeed, alterations in vascular responsiveness often precede an increase in arterial stiffness, a prognostic index of cardiovascular health (11). Given its importance, numerous studies investigated how to ameliorate vascular responsiveness through both pharmacological and non-pharmacological approaches (12–16). The non-pharmacological approach often involves endurance training performed with large muscle mass (e.g., running, jogging, or cycling), and has received the most attention and endorsement in terms of effectiveness in improving vascular responsiveness (12–15).

In recent years, endurance training involving small muscle mass, such as single-leg knee extension training (SLKE) has also received growing interest as a method for improving vascular responsiveness. SLKE is being employed to maximize vascular adaptation due to its ability to circumvent central limitations and sympathetic restraint of limb blood flow (Q̇) (17). Endurance exercise using small muscle mass was also shown to improve local circulation, oxygen extraction, and muscles’ force (18–20). The increased metabolic demand of the muscles involved, such as a higher oxygen requirement from the contracting muscles and the need to remove some exercise-induced metabolites, matches the increment in Q̇ and therefore in shear rate ($\dot{Y}$), the frictional force acting on the vessel’s inner lumen (i.e., the mechanical stress acting on the endothelial cells) (21–23). Additionally, although with conflicting results (18), a systemic effect on vascular responsiveness (i.e., on the arteries not directly involved in the exercise) was also reported (22). Despite its greater feasibility compared with endurance training, small muscle mass endurance exercise might still remain precluded to people with limited mobility.

On this path, an increasing body of literature has focused on the effects of passive exercise methods (e.g., passive static stretching) as a means to maintain or even improve vascular responsiveness, reporting very interesting and encouraging results (24, 25). For example, 60 sessions of passive static stretching performed over 12 weeks significantly improved vascular responsiveness at both local and systemic levels (24). A plausible explanation for the effects of passive static stretching at the local level may depend on the repetitive increases and decreases in $\dot{Y}$ occurring during the elongation and relaxation phases of passive static stretching, which can trigger a chain of reactions that may lead to higher endothelial nitric oxide (NO) bioavailability, thus increasing the vascular responsiveness (9, 18, 26). At the systemic level, the continuous stretch-induced fluctuations in $\dot{Y}$ could generate a continuative alternance of vasoconstriction and vasodilation, and consequently a possible stimulus towards the autonomic nervous system in controlling the Q̇ distribution (11, 24, 27).

Although not uniquely reported in the literature, the fluctuations in $\dot{Y}$ may also play an important role during endurance small muscle mass exercise as the SLKE (18). Although the mechanism might be similar, the extent of its effects on both local and systemic vascular responsiveness when performing SLKE or passive static stretching training (PST) was assessed in separate studies and has not been directly compared in the same population. Should the results be comparable, people with limited mobility may benefit from performing PST, definitely less demanding and more feasible than SLKE. On these bases, the purpose of the present study was to evaluate the effects of SLKE vs PST on the vascular responsiveness at both local and systemic level determined by Doppler ultrasound using the single passive limb movement (sPLM) and flow-mediated dilation (FMD) test. It was hypothesized that both SLKE and PST would improve vascular responsiveness, with the former inducing greater adaptations.

**MATERIALS AND METHODS**

**Participants**

Figure 1 illustrates the study flowchart. A total of 42 low-active healthy adults volunteered to participate in the study (age range: from 20 to 30 years). The level of physical activity was determined by the Italian version of the International Physical Activity Questionnaire (28). Exclusion criteria were: (i) presence of neurological, vascular, and musculoskeletal impairments at the level of the lower and upper limbs; (ii) being on pharmacological therapy related to neural and/or vascular response, including hormonal contraceptives and oral supplements; (iii) being a current or former smoker; (iv) systolic arterial pressure higher than 140 mmHg and diastolic higher than 90 mmHg; and (v) having an irregular menstrual cycle (shorter than 26 or longer than 35 days) up to 3 months before the beginning of the study, (vi) contraindications to joint mobilization and; (vii) being regularly involved in aerobic or PST program. All participants supplied written, informed consent after being informed about the aims of the study and the potential risks derived from tests and methods. The Institutional Review Board of the Università degli Studi di Milano approved the study (CE 27/17). The study was registered at ClinicalTrial.gov (ID: NCT04758754) and performed by the principles of the Helsinki Declaration. The researchers who analyzed the data were blinded to group allocation.

-Figure 1 about here-

**Study design**

Before testing procedures, participants underwent a preliminary session during which they familiarized themselves with the dynamic knee extension ergometer and with the procedure to identify the maximum isometric voluntary contraction (MVC) of the knee extensor muscles of both limbs. During this visit, the sPLM and the FMD tests (see below for a full explanation of the procedures) were performed on each participant. At the end of the tests, the ultrasound probe position for testing was marked on a transparency sheet, together with some skin landmarks (moles, scars, angiomas, etc.). The sPLM and FMD outcomes obtained during the familiarization and the pre-training experimental session were used to calculate intersession reliability. Knee flexion ROM was also measured.

All measurements were taken at the beginning (PRE) and after 8 weeks (POST) of SLKE or PST training. The participants were tested at the same time of the day in a climate-controlled laboratory [temperature 20(1)°C and relative humidity 50(5)%] to minimize confounders due to circadian rhythms. For females, the tests were assessed on the same day of the menstrual cycle [early follicular phase days 3(3)]. Female participants recorded their menstrual cycle in a personal diary throughout the study, which was used to assess the early follicular phase and allowed the subjects to be tested on the same menstrual day pre-and -post-training. On the test days, participants came to the laboratory after fasting overnight, abstaining from caffeine and other similar substances for at least 12 h, and not taking part in heavy exercise for at least 48 h before the tests. Post-training assessments were performed 48 h after the last training session. As in a previous study, this period was observed to possibly prevent biases in measurements introduced by the acute effects of the last training session (18).

**Measurements and data analysis**

Measurements were performed bilaterally in all groups for the lower and upper limbs. Data are presented as the average between the two limbs.

*Range of motion (ROM).* To check the changes in knee joint ROM, a bi-axial angle transducer (TSD 130A, Biopac System, Goleta, CA, USA) was used. The angle transducer was placed with one axis on the external condyles of the knee joint and the other on the external face of the fibula.

*Maximum isometric voluntary contraction (MVC).* Knee extensors MVC was measured with the participants sitting on an ergometer with the hip and the knee flexed at 90° and firmly secured at the ankle level by a Velcro® strap (Velcro Industries Inc., Willemstad, Netherlands Antilles) to a load cell (mod. SM-2000N operating linearly between 0 and 2000 N; Interface, Crowthorne, United Kingdom) for the force signal detection. After a standardized warm-up (10 × 2-s contractions at 50% MVC previously determined during familiarization), three MVC attempts were performed. The participants were instructed to push as fast and hard as possible for 3 s. Each MVC attempt was interspersed by 3 min of recovery. The force signal was driven to an A/D converter (mod. UM 150, Biopac, Biopac System Inc., Santa Barbara, CA, United States), sampled at 1000 Hz, and stored on a personal computer. The maximum force value recorded during tests was considered MVC and was inserted into the data analysis.

*Maximum Work Rate (Ẇmax).* The maximum work rate (Ẇmax) was determined by an incremental square wave test on a dynamic knee extension ergometer. The test was performed while sitting on an adjustable chair to fit body sizes of different dimensions. Both knees were flexed at 90°, with the ankle of the dominant limb connected to a bicycle ergometer pedal arm by a rigid bar. The concentric phase occurred actively from 90° of the knee to full extension, while the eccentric phase was completely passive, driven by the flywheel momentum. The mechanical brake applied to the ergometer and the pedal frequency were measured to determine the mechanical power output. The mechanical friction, i.e., the force applied to each revolution, was measured by a force transducer (mod. SM-100 N, Interface, Crowthorne, UK), while the pedal frequency was determined by a magnetic transducer integrated into the cycle ergometer (mod. 839E, Monark, Vansbro, Sweden). Both the force and the pedal frequency signals were amplified (gain × 100) and acquired by a personal computer after an A/D conversion (model UM150, Biopac System, CA, US) at a sampling rate of 1 kHz. The square wave test started with a first workload of 15 W. Loads increased by + 5W until exhaustion (29). Each load was kept for 3 min, with 5 min recovery in between. The load of the last completed step was considered as Ẇmax. A non-invasive impedance cardiograph device (Physio Flow®, Manatec Biomedical, Paris, France) was used to assess the cardiac output (Q̇_T_), stroke volume (q), and heart rate (HR). At rest and during the test, pulmonary oxygen uptake (V̇O_2_) was detected on a breath-by-breath modality by gas analyzers (mod. Quark b^2^, Cosmed, Rome, Italy). The system was calibrated before each test with gas mixtures of known concentrations (16% O_2_; 5% CO_2_; balance N_2_). Data were averaged on the last 60 s of baseline and the last 30 s of the last workload. The average V̇O_2_ matched to Ẇmax was considered as peak V̇O_2_ (V̇O_2_p).

*Muscle thickness.* Participants lay in a supine position on an examination bed with their knees in the anatomical position. By real-time B-mode ultrasonography with a 9-MHz linear (mod. Logiq-7, General Electric Medical Systems, Milwaukee, WI, US) three images were taken on the center of vastus lateralis, vastus intermedius, and rectus femoris muscle belly using sagittal plane ultrasound scans, halfway between the lateral epicondyle of the knee and the greater trochanter. The site was clearly marked on the skin with indelible ink and further outlined on tracing paper, to supply a standardized measurement site and ensure that assessments at all measurement points were taken from the same external site. Minimal pressure was applied on the skin during scans, which were saved and analyzed offline with publicly available imaging software (ImageJ 1.43b; NIH, Bethesda, MD, USA). Muscle thickness, defined as the perpendicular distance between the superficial and deep aponeurosis, was measured in the proximal, mid, and distal regions in each ultrasound image, and the average of the 3 values was used for further analyses.

*Single passive limb movement (sPLM).* sPLM was performed by the recommended procedures (30). Sitting on a chair, subjects rested in the upright-seated position for 10 min before starting the data collection and remained in this position until the end of the test. The sPLM protocol consisted of 60 s of baseline peripheral hemodynamic data collection, followed by single passive knee flexion and extension of 1 s, after which the leg was kept fully extended for the remaining 59 s for the post-movement data collection. PLM was performed by a member of the research team, who moved the subject’s lower leg through a 90° range of motion at 1 Hz. Throughout the test, measurements of arterial blood velocity and vessel diameter were performed in the common femoral artery of the passively moved leg, distal to the inguinal ligament and proximal to the deep and superficial femoral bifurcation by Doppler ultrasound (mod. Logiq-7, General Electric Medical Systems, Milwaukee, WI, US). After being positioned to an insonation angle of 60° or less, a 9-MHz linear array transducer was used to measure the mean blood velocity. The sample volume was centered and size-maximized according to the vessel’s diameter (31). Femoral artery Q̇ (Q̇_fem_) was calculated by using data of arterial diameter and mean blood velocity as:

Q̇ (ml⋅min^-1^) = mean blood velocity ⋅ π ⋅ (diameter /2)^2^ ⋅ 60

The cumulative Q̇_fem_ was integrated (area under the curve, AUC) using the trapezoidal rule and then calculated. Reliability analysis for sPLM was previously reported (18, 24).

Before the sPLM, the systolic and diastolic arterial pressure was measured at rest by a digital sphygmomanometer (mod. HEM-907, Omron, Hoofddorp, The Netherlands). The mean arterial pressure was therefore calculated (MAP)

*Flow-mediated dilatation (FMD).* FMD was measured at the brachial artery according to recommended procedures (32). Before FMD, the participants lay supine for 20 min to restore baseline cardiovascular values. An arterial pressure cuff was placed on the forearm immediately distal to the olecranon process to generate an ischemic stimulus in the brachial artery when inflated. Following baseline assessment, the blood pressure cuff was inflated to 250 mm Hg for 5 min. Arterial diameter and mean velocity recordings resumed at baseline, 30 s before cuff deflation, and continued for 120 s post-deflation, as described elsewhere (10, 19, 32). A 15-MHz linear array transducer attached to a high-resolution ultrasound machine was used to image the brachial artery in the distal third of the upper arm. When an optimal image was obtained, the probe was held stable and longitudinal in B-mode, and images of the lumen-arterial wall interface were acquired. Continuous Doppler velocity was measured simultaneously, and the data were collected using the lowest possible insonation angle (<60°). The FMD data were exported in AVI format and analyzed using commercially available software (Brachial Artery Analyzer for Research, Medical Imaging Applications, LLC, Coralville, IA, US). An experienced operator double-checked the analysis of the data. FMD was quantified as the maximal change in an artery diameter after cuff release, expressed as a percentage increase above baseline (%FMD):

%FMD = (peak diameter – baseline diameter)/ baseline diameter × 100.

Brachial artery blood flow (Q̇_brac_) was calculated as described for sPLM measurement.

Brachial artery $\dot{Y}$ was calculated post-cuff release with the equation:

$\dot{Y}$ (s^−1^) = 8 ⋅ mean velocity/diameter

The cumulative $\dot{Y}$, corresponding to the reactive hyperaemia post-cuff release (total $\dot{Y}$ from cuff release to time-to-peak, $\dot{Y}$ AUC) was calculated using the trapezoidal rule. The $\dot{Y}$ AUC reflects the amount of mechanical stimulus applied to the endothelium during cuff release hyperemic response until time-to-peak. Given that FMD is primarily dependent on endothelial response to mechanical stimuli, the FMD was divided by the $\dot{Y}$ AUC (FMD/$\dot{Y}$) (21). Reliability analysis for FMD was reported previously (18, 24).

*Acute hemodynamic measurements*. To evaluate the possible difference in the brachial artery vascular response between the exercise conducted without the use of the upper limbs and that with the use of the upper limbs (a condition similar to that used during training), the $\dot{Y}$ and the MAP were assessed in all the study participants (i) during an acute 5-min SLKE at 70% Ẇmax (representing a typical workload intensity within the training) and (ii) during a single bout PST of the knee extensor muscles, both performed with and without the use of the upper limb muscles (see Fig. 2 for a photographic representation of the experimental set up). These evaluations were performed in different sessions separated by at least 72 hours. During the two acute evaluations, the artery’s diameter and the mean velocity were continuously measured from the brachial artery of the right arm. i.e., the arm stabilizing the stretched muscle during PST administration, and the participant’s position on the ergometer during SLKE. The mean $\dot{Y}$ during the 5-min single-leg exercise and the 5 elongations and relaxation phases of the passive stretching bout was calculated as in the FMD tests (Doppler ultrasound, mod. Logiq-7, General Electric Medical Systems, Milwaukee, WI, US). Concomitantly, the MAP was determined on a beat-by-beat basis using a finger photoplethysmography device (FinometerPro; Finapres Medical Systems, Amsterdam, The Netherlands), with the photoplethysmography cuff placed on the third finger of the left hand. The height adjustment and reference sensors were positioned following the manufacturer’s instructions. The blood pressure signal was calibrated by the procedure indicated by the manufacturer.

The $\dot{Y}$ and the MAP were calculated over time windows of 5 s and then averaged every minute. Only for the measurements relating to stretching, the $\dot{Y}$ and the MAP were also calculated considering separately the elongation phases (average over 45 s) and the relaxation phase (average over 15 s), due to a different behavior that two variables arise during the two phases (24).

During the single-leg knee exercise, the participants sat on the same ergometer in the test for Ẇmax determination. The assessment started with a 3-min measurement of baseline condition, then measurements were made continuous throughout the 5-min exercise (18). Exercise was performed with and without stabilizing the position of the participant on the ergometer.

During the passive stretching assessment the participants rested in a supine position for 20 min before starting the data collection and remained in this position throughout the entire duration of the data collection (33). Passive stretching protocol consisted of 5 min of resting baseline followed by passive static elongations for 45 s and passive relaxations for 15 s, repeated five times. During the entire PST protocol, the muscles were stretched by the same operator up to a point of discomfort. The knee joint angle was continuously recorded using a biaxial angle transducer (mod: TSD 130A; Biopac Systems, Santa Barbara, CA, US). Force output between the passively stretched leg and the operator's arms was recorded during the protocol by a load cell (model SM-2000 N; Interface, Crowthorne, UK). Specifically, the load cell was positioned 5 cm above the ankle and a member of the research team pushed perpendicularly the load cell to stretch the leg extensor for 45 s. The passive force output during the 45 s of the consecutive flexion of the PS protocol was displayed on a computer screen to keep constant the passive force throughout the elongations. In the second session the measurements were performed with the participant positioned as in the Figure 2 panel B, which is the stretching exercise in which the participant position was more stable and that could have induced a greater activation of the upper limb muscles. Briefly, the participant was on one knee with the contralateral foot resting on the ground. From this position, the supporting knee was flexed, and the ipsilateral hip was extended. The right arm maintained actively the elongation. The forearm of the left limb was placed on a table to increase the stability of the position during the measurement, and to allow the positioning of the finger photoplethysmography device without any constriction.

During SLKE and PST the right arm was involved to stabilize the position of the participant on the ergometer in SLKE, and to keep the elongation of the muscle involved in the stretching exercise in the PST while the left arm was located on a support to allow the positioning of the finger photoplethysmography device without any constriction. A certain influence on the brachial artery vascular response due to the activation of the muscles of the upper limbs could therefore have occurred.

-Figure 2 about here-

**Training**

Both SLKE and PST were performed on both limbs and had a similar duration of 100 min × week for a total duration of 800 min (400 min each limb). To avoid overloading at knee extensors and knee joint level, SLKE was performed 3 times × week, with a single session duration of 33 min and 20 s. PST was performed 5 times × week, with a single session duration of 20 min. To enhance compliance, training sessions were held at different times of the day (morning and afternoon) at the University Sports Centre gym. Sessions were supervised by expert operators who checked attendance, correct exercise execution, and intensity. Participants not attending at least 80% of classes were excluded from the study, and a new participant was recruited to substitute the dropout. The CTRL group received no training.

*Passive static stretching training (PST).* PST included 8 weeks of PS training, 5 sessions × week (40 sessions in total). Each PST session lasted 20 min and included two exercises for the knee extensor muscles (Fig. 3, panels A and B give an example of the PST exercises): 45 s elongation and 15 s recovery in the resting position; the set was repeated five times (24). The muscle elongation was maintained by the participant at the 80% of the point of discomfort (24).

*Single leg knee extension muscles training (SLKE).* Participants underwent an 8-week SLKE (3 sessions × week, 24 sessions in total) involving both legs, one at a time. SLKE was performed on the same ergometer used for Ẇmax assessment, which allows participants to train only leg extensor muscles of a single limb (Fig. 3, panels C1 and C2). The leg starting order was balance among training sessions. Workloads ranged from 70% to 95% Ẇmax, with an average session duration of 16 min and 40 s for each leg (see Table, Supplemental Digital Content 1, Characteristics of each single-leg knee extension training session, http://links.lww.com/MSS/C446). The session’s workload was readjusted every 2 weeks reassessing a new Ẇmax.

-Figure 3 about here-

**Statistical analysis**

Statistical analyses were performed using a statistical software package (IBM SPSS Statistics v. 26, Armonk, NY, USA). Based on previous research (18, 24) for this study we used changes in Q̇ as main outcomes, a two-way analysis of variance (ANOVA; within-group factor: time; between-groups factor: intervention) in the statistical approach, with an α level of 0.05 and a required power (1-β) of 0.80; the desired sample size computed using statistical software (G-Power 3.1, Dusseldorf, Germany) resulted in 36 participants.

The Shapiro-Wilk test was used to check the normal distribution of the sampling. A three-way ANOVA for repeated measures [RM, within-group factor: time, 6 levels (baseline + 5 phases), between-groups factor: condition, 2 levels (with or without active arm); phase (elongation or relaxation)] was applied to check for differences in $\dot{Y}$ and MAP during the elongation and the relaxation phases occurring in an acute passive stretching bout. A two-way mixed-model analysis of variance (ANOVA) [within-group factor phase: 6 levels (baseline + 5 phases; between-groups factor: group, 4 levels (single-leg knee extension exercise without active arm, single-leg knee extension exercise with active arm, passive stretching without active arm, and passive stretching with active arm)] was applied to check for differences in $\dot{Y}$ and MAP during the 5-min of single-leg knee extension exercises the acute passive stretching bout. To calculate the difference in changes between the groups, analysis of covariance (ANCOVA) was performed, entering the baseline (PRE) values as a covariate.

A two-way mixed-model analysis of variance (ANOVA) [within-group factor: time, 2 levels (PRE, POST); between-groups factor: group, 3 levels (SLKE, PST, and CTRL)] was used to assess the significant effects of training. To calculate the difference in changes between the groups, analysis of covariance (ANCOVA) was performed, entering the baseline (PRE) values as a covariate. Multiple comparisons were performed applying Bonferroni’s correction. The ANOVA effect size was evaluated with partial eta squared (*η_p_^2^*) and classified as follows: <0.06: *small*; if, 0.06-0.14: *medium*; and >0.14: *large* (34). The Hedge’s *g* effect size with CI_95%_ was also calculated (<https://www.cem.org/effect-size-calculator>) and interpreted as follows: 0.00-0.19: *trivial*; 0.20-0.59: *small*; 0.60-1.19: *moderate*; 1.20-1.99: *large*; ≥2.00: *very large* (35)*.* Data are presented in the mean (SD). Statistical significance was set with *P*<0.05.

**RESULTS**

**Participants’ compliance**

Thirty-six low-active participants [18 females and 18 males, age 22(1) years, body mass 68(4) kg, stature 1.67 (0.10) m, mean (standard deviation) (SD)] were enrolled and randomly assigned to one of three groups, balancing the sample for sex: SLKE [n = 12 (6F/6M), age 22(2) years, body mass 69(3) kg, stature 1.68(0.10) m]; PST [n = 12 (6F/6M), age 23(1) years, body mass 68(4) kg, stature 1.70(0.10) m], and control group [no training, CTRL: n = 12 (6F/6M), age 22(3) years, body mass 69(2) kg, stature 1.69(0.09) m]. Participants’ attendance was about 93% (268/288 training sessions) and 89% (427/480 training sessions) for SLKE and PST, respectively. Two participants for SLKE and one for PST dropped out throughout the study because of injury (unrelated to the training protocols) and lack of time. They were immediately replaced to maintain the required sample size.

**ROM, MVC, Ẇmax, muscles thickness and cardiopulmonary parameters**

Table 1 provided the PRE-POST changes in ROM, MVC, Ẇmax, muscle thickness, and in rest and peak values of Q̇_T_, q, HR, MAP and V̇O_2_. ANOVA disclosed a significant *time* × *group* interaction in ROM, MVC, Ẇmax, muscle thickness and V̇O_2_p. ROM increased only in PST in POST by 6(3)% (*g*: 2.18 (1.17 − 3.20, *P*<0.001). Conversely, MVC, Ẇmax, muscle thickness and V̇O_2_p increased only in SLKE by 23(2)% [*g*: 1.67 (0.74 − 2.60), *P*<0.001], 24(22)% [g: 0.84 (0.00 − 1.67), *P*<0.001], about 6(4)% (g from 0.45 to 0.84, *P* from 0.001 to <0.001), and 15(13)% [g: 0.85 (0.01−1.69), *P*<0.001], respectively. MAP decreased in SLKE and in SPT by -12(4)% [g: -2.09 (-3.09 −1.10), *P*<0.001] and -7(49% [g: -0.99 (-1.84 − -0.14), *P*<0.001] No significant changes occurred in CTRL. ANCOVA disclosed significant differences in the increase for ROM in SPT compared to SLKE and CTRL (g: 1.28 and 0.97, *P*<0.001, respectively) and for MVC (g: 1.69 and 1.68, *P*<0.001), Ẇmax (g: 4.99 and 4.98, *P*<0.001), muscle thickness (g from 0.42 to 1.52, *P* from 0.004 to <0.001), and V̇O_2_p (g: 0.76 and 0.77, *P* = 0.001) in SLKE compared to SPT and CTRL. MAP decreased more after SLKE than PST and CTRL.

-Table 1 about here-

**sPLM**

Table 2 and Figure 4 demonstrated the PRE-POST difference in the sPLM variables. ANOVA disclosed significant *time* × *group* interactions in all the variables. Rest, peak, and ΔQ̇_fem_ increased in SLKE and PST by 35(13)% and 7(5)% [SLKE g: 0.91 (0.07−1.75, *P* = 0.01), PST g: 0.51 (0.30 − 1.33, *P* = 0.02], by 47(4)% and 14(2)% [SLKE g: 3.71 (2.39−5.03), *P*<0.001, PST g: 2.26 (1.24 − 3.29, *P*<0.001], and by 54(7)% and 20(2)% [SLKE g: 2.72 (1.61−3.83), *P*<0.001, PST g: 2.43 (1.38 − 3.49, *P*<0.001]. AUC increased in POST in SLKE by 60(11)% [g: 2.06 (1.07 − 3.05), *P*<0.001] and in PST by 11(3)% [g: 0.76 (0.07 − 1.59), *P* = 0.003]. No significant changes occurred in CTRL. ANCOVA evidenced increments in all sPLM variables larger in SLKE than in PST and CTRL (g from 0.24 to 2.95, *P* from 0.03 to <0.001).

-Table 2 and Figure 4 about here-

**FMD**

Table 3 and Figure 5 illustrates the PRE-POST differences in the FMD variables. Except for rest diameter and $\dot{Y}$, ANOVA found significant *time* × *group* interactions in all the other variables. Peak diameter increased in POST in SLKE and PST by 6(2)% [*g*: 0.54 (0.26 − 1.06), *P* = 0.007] and 4(1)% [*g*: 0.54 (0.27 − 1.36), *P*< 0.001], respectively. In POST %FMD increased by 12(2)% [*g*: 0.68 (0.14 − 1.51), *P*<0.001] and 11(1)% [*g*: 0.83 (0.04 − 1.66), *P*<0.001] in SLKE and PST. FMD/$\dot{Y}$ increased in POST by 18(2)% [*g*: 0.66 (0.16 − 1.49), *P*<0.001] and 17(2)% [*g*: 0.67 (0.15 − 1.49), *P*<0.001] in SLKE and PST. In POST, AUC increased by 14(7)% [*g*: 0.60 (0.22 − 1.42), *P*<0.001] and 10(3)% [*g*: 0.66 (0.16 − 1.48) *P*<0.001] in SLKE and PST, respectively. No changes occurred in CTRL in any variables. Remarkably, ANCOVA disclosed similar PRE-POST increments in SLKE and PST in all the FMD variables, which were significantly larger compared to CTRL (g from 0.64 to 1.84, *P* from 0.002 to <0.001).

-Table 3 and Figure 5 about here-

**Acute hemodynamic measurements**

Table 4 panel A demonstrated the acute changes in the mean $\dot{Y}$ at brachial artery level and the MAP during the 5-min of single-leg knee extension and the single bout of passive stretching. Two-way ANOVA reported no time × condition interaction for the mean $\dot{Y}$ and MAP during the single-leg knee extension and the passive stretching bout. Main effects for time were retrieved for the mean $\dot{Y}$ and MAP during the single-leg knee extension and the passive stretching bout. ANCOVA disclosed significant differences in the increases in MAP between the single-leg knee extension and the passive stretching (*g* from 1.13 to 4.43, *P* from 0.02 to <0.001). On the contrary, no differences were shown for changes in $\dot{Y}$ between the different conditions.

During the 5-min of single-leg knee extension, $\dot{Y}$ without active arm increased compared to the baseline from the 2^nd^ to the 5^th^ minutes by on average 28(20)% (*g* from 0.64 to 2.96, *P* from 0.017 to 0.008), and with active arm from the 1^st^ to the 5^th^ minutes by on average 26(11)% (*g* from 0.59 to 1.15, *P* from 0.027 to 0.005). During the 5-min passive stretching bout, $\dot{Y}$ without active arm increased compared to the baseline in the 1^st^, 3^rd^ and, 4^th^ minute by on average 13(3)% (*g* from 0.35 to 0.82, *P* from 0.008 to <0.001), and with active arm from the 2^nd^ to the 3^rd^ minutes by on average 16(4)% (*g* from 0.32 to 0.90, *P* from 0.016 to 0.019).

MAP increased only during the single-leg knee extension from the 1^st^ to the 5^th^ minutes in both conditions, by on average 30(16)% (*g* from 1.41 to 4.06, *P* from 0.039 to 0.001), and 23(11)% (*g* from 4.18 to 4.56, *P* from 0.002 to <0.001), without and with the active arm, respectively.

Table 4 panel B reported the changes in $\dot{Y}$ and MAP during the single-leg knee extension and the elongation and relaxation phase of passive stretching in the two conditions. Three-way ANOVA reported no time × condition × phase and no time × condition interaction, but a significant time × phase interaction. No between-conditions differences were found. During the elongation phase, $\dot{Y}$ decreased compared to the baseline from the 2^nd^ to the 5^th^ elongation by on average -14(8)% (*g* from -1.03 to -0.79, *P* from 0.017 to 0.001) without active arm, and during the 4^th^ and 5^th^ elongation with active arm [ -8(7)%, *g* = -0.47 and -0.65, *P* from 0.03 to 0.01]. MAP without active arm increased from the 2^nd^ to the 4^th^ elongation by -4(2)% (*g*: from -0.61 to -0.31, *P* from 0.02 to 0.007), and MAP with active arm from the 2^nd^ to the 5^th^ elongation by -5(2)% (*g* from -0.80 to -0.45, *P* from 0.04 to 0.02).

During the relaxation phase, the mean $\dot{Y}$ without active arm increased during the five maneuvers by 30(12)% (*g* from 1.26 to 1.60, *P* from 0.017 to <0.001), and with active arm by 33(11)% (*g* from 0.87 to 2.04, *P* from 0.04 to <0.001). MAP without active arm decreased from the 2^nd^ to the 5^th^ maneuver by -4(2)% (*g* from -0.80 to -0.45, *P* from 0.04 to 0.02), and with active arm by -5(2)% (*g* from -0.52 to -0.34, *P* from 0.04 to 0.01).

-Table 4 about here-

**DISCUSSION**

This study evaluated the local and systemic vascular responsiveness adjustments induced by an 8-week SLKE or PST intervention. In line with our hypothesis, SLKE induced greater improvements in local vascular responsiveness than PST. Surprisingly, SLKE and PST led to similar vascular responsiveness improvements at the systemic level. While locally the action of multiple concomitant mechanisms triggered a greater increase in vascular responsiveness after SLKE, at the systemic level, the similar changes in $\dot{Y}$ produced by the two training regimens was likely the reason for the equal increases in vascular responsiveness.

**Preliminary consideration**

Similar to previous studies (18, 24, 36), both SLKE and PST have shown their effectiveness. An improvement in peak V̇O_2_, Ẇmax, and MVC of the knee extensor muscles was indeed already reported after SLKE of equal duration (but lower intensity) in a group of moderately active young people (18). These adaptations occurred with no changes in heart hemodynamics due to the minimum taxing of this small muscle mass exercise paradigm on central factors. These findings are also in line with previous reports in health and disease (37–39), indicating that this specific training paradigm can induce improvements in peripheral convective and diffusive oxygen transport aspects without detectable changes in central hemodynamics.

An increase in ROM without any change in knee extensor muscle MVC occurred after the 8-week PST. These findings are in line with a previous investigation, in which an increase in ROM coupled to a preserved MVC of the plantar flexor muscles was reported after 12-week PST (36).

Under a technical point of view, although we followed the recommended technique and analysis to determine %FMD, the %FMD values are quite a bit higher than typically expected (40). The involvement of young, healthy, and sedentary participants may possibly explain the higher % FMD values reported here.

**SLKE and PST effects on local vascular responsiveness**

sPLM response increased after both SLKE and PST but with ameliorations that were from 4- to 5-folds larger in the former than in the latter training modality. Previous investigations indicated that sPLM response can provide important insights into vascular responsiveness, being more representative of a microvascular assessment and closely related to local vasodilatory factors (30, 41). The most representative sPLM variables involve: (i) the peak flow; (ii) the change from baseline to peak flow (∆ peak flow); and (iii) AUC (30, 42). As previously reported, all these parameters are related to NO bioavailability (42). Considering vascular tube length and blood viscosity relatively constant, and applying Poiseuille’s law, sPLM-induced hyperemia may be driven by two main factors: an increase in perfusion pressure and peripheral vasodilation (i.e., drop in the peripheral resistance). Our data reveal an increase in the *Q̇_fem_* at rest after both SLKE and PST, together with a lower MAP. These findings may be ascribed to a reduction in total peripheral vascular resistance that led to an increased muscle perfusion. Indeed, sPLM causes the mechanical deformation of the vessels determining vasoactive-substances release, among which NO (43, 44), which, in turn, results in the dilatation of the vascular bed (31). Therefore, considering the nature of this hyperemic response, we can ascribe the sPLM-induced hyperemia, at least in part, related to an enhanced NO-bioavailability (30, 45). The oscillations in Q̇ during the two interventions and, in turn, in $\dot{Y}$, could have resulted in an important stimulus to the endothelial cell membrane that triggered a cascade of signaling events favoring NO bioavailability (18, 24, 27).

As previously suggested, the amplitude of vascular responsiveness improvement seems to be $\dot{Y}$-magnitude dependent (9). Our results implied that the $\dot{Y}$ in the femoral artery produced during SLKE was higher compared to PST, thus producing in the former a larger improvement in local vascular responsiveness. Greater $\dot{Y}$during SLKE would be the effect of greater metabolic demand by the muscles involved with exercise. A greater increase in local vascular responsiveness after SLKE compared to PST could be further explained by the increase in muscle thickness that was induced only after SLKE. An increased muscle volume leads to a greater microcirculation, a phenomenon that has an important impact on the response of the sPLM (8, 46). Instead, regarding the improvement of local vascular responsiveness after PST, an additional mechanism that should be considered is the possible change in vessel tortuosity. Indeed, the enhancement of the microvasculature, coupled with a lower MAP, could be explained by a reduction in total peripheral vascular resistance. Vessel tortuosity is linked to this resistance in large and small blood vessels and to Q̇ distribution (47). Vessel tortuosity describes how twisted the capillaries are and how many turns and bends they have, on which basis a physiological index of the capillary extension reserve is defined (47, 48). Changes in muscle sarcomere length reduce significantly the capillary extension reserve (i.e., tortuosity) (47, 48). Extensive muscle lengthening (as in PST) results in repeated cycles of vessel elongation and compression, leading to a reduction in vessel resistance and capillary diameter, as well as in Q̇ and O_2_ supply (48). The acute vessel distortion is an important stimulus for long-term vascular adaptations induced by PST. While our study did not supply direct evidence for changes in vessel tortuosity, its reduction following PST cannot be ruled out. Lastly, the continuous oscillations of Q̇, and therefore of $\dot{Y}$, induced by active and passive exercise have been advocated as possible mechanisms for the increase in the levels of vascular endothelial growth factor protein, in endothelial cell proliferation rate, and in eNOS messenger RNA levels, all aspects that can improve vascular responsiveness (49, 50). The lack of metabolic activation of the muscles during stretching compared to active exercise could justify the differences seen in local vascular responsiveness reported here. Although vascular endothelial growth factor protein levels, endothelial cell proliferation rate, and endothelial NOS messenger RNA levels before and after training were not evaluated in the present study, their possible contribution to local vascular responsiveness enhancement may play a role.

**SLKE and PST effects in systemic vascular responsiveness**

Eight weeks of SLKE and PST led to an improvement also in brachial artery vascular responsiveness, an artery distal from the application point of the mechanical stimulus. The acute measurement of Q̇ has shown that significant changes in $\dot{Y}$ at the level of the brachial artery occurred during both active and passive exercise, which were accompanied by a concomitant adaptation of the MAP. Although some activation of the upper limb muscles occurred during the two training modalities (during SLKE to stabilize the participant in the ergomenter and during PST to keep in elongation the stretched muscles), the acute changes in $\dot{Y}$and MAP were similar between the passive condition (i.e., without active arm) and the condition with the intervention of the upper limb muscles (i.e., with active arm). This suggests that the contribution of the activation of the upper limb muscle to the improvement of the brachial artery vascular responsiveness could have been minimal and therefore the improvements in the brachial artery could be mainly due to the changes in $\dot{Y}$ induced by the two training modalities.

The continuous changes in $\dot{Y}$ during the two training methods may have contributed to an improvement in vascular responsiveness. Surprisingly, the changes in $\dot{Y}$ were not different between the two interventions, suggesting a systemic response not proportional to the muscular effort required during active and passive exercise. An increase in vascular responsiveness following SLKE and PST is in line with previous reports, which observed an improvement in some central mechanisms of Q̇ control as a possible explanation for the systemic ameliorations indicated by the FMD increment in the brachial artery. It should be kept in mind that the FMD response is strongly correlated with the dilation capacity at the coronary level (3). Therefore, the improvement in systemic vascular responsiveness detected in the present study could also have important implications on cardiovascular risk.

The present study did not allow to evaluate whether the vascular responsiveness ameliorations retrieved at the brachial artery level could have been induced by circulating levels of vascular endothelial growth factor, likely triggered by the active and passive remote exercise training. Although further studies will be needed to evaluate this mechanism, its possible involvement in explaining systemic changes in vascular responsiveness cannot be ruled out. Contrary to the present work, a previous investigation did not reveal a systemic vascular responsiveness improvement following 8 weeks of SLKE (18). A larger sample size included in the present investigation could possibly justify the differences that emerged between the two studies.

**Study limitations**

The present study comes with some limitations. First, although the effect of the upper limb muscles in increasing the $\dot{Y}$ in the brachial artery was minimal, in the long term it might have improved the brachial artery responsiveness due to local instead of systemic mechanisms only. Second, the sPLM and FMD represent the function of two distinct types of vessels and are not representative of similar mechanisms, being sPLM response correlates to the resistance artery or microcirculatory function, while FMD represents the function of the conduit artery. However, they could provide evidence for an improved overall vascular responsiveness at the local (sPLM) and systemic (FMD) level. To have a clearer evaluation of the effect of training on the local and systemic blood flow control-mechanisms, the last should involve conduit arteries or resistance arteries of both the upper and lower limbs. Third, direct assessment of muscle sympathetic nerve activity, NO bioavailability, and vascular endothelial growth factor protein levels would have confirmed possible training-induced modulation of sympathetic vessel tone and endothelial function. The lack of a direct assessment of these measurements did not allow us to establish a clear balance between central and local mechanisms underlying the positive vascular responsiveness changes. Forth, the evaluation of local and systemic vascular responsiveness parameters after a follow-up period could have highlighted any differences between the two interventions in the persistence of their effects. Unfortunately, due to the COVID-19 pandemic situation and the limitations imposed on the laboratory’s attendance, it was not possible to plan a further evaluation related to the follow-up. Lastly, the systemic vascular effects reported in this study refer only to the comparison between PST and SLKE and do not give indications with respect to the systemic vascular adaptations obtainable with endurance training involving large muscle masses, such as cycling and running (which should induce larger systemic adaptations compared to PST).

**CONCLUSIONS**

Despite involving similar muscle mass (knee extensor muscles), larger increases in local vascular responsiveness after active (SLKE) than after passive training (PST) occurred. Surprisingly, similar systemic vascular responsiveness improvements (similar increases in FMD parameters at the level of the brachial artery) were observed after both interventions. While at the local level the greater variations of Q̇ (and therefore of $\dot{Y}$) during SLKE may have led to a more evident vascular responsiveness improvement. On the contrary, FMD findings would suggest that the vascular responsiveness at the systemic level was not affected in a directly proportional way to the muscular demands. From a practical point of view, these results would indicate how also a passive method (PST) can trigger significant improvements in systemic vascular responsiveness. This would be particularly crucial given the importance of maintaining or even improving vascular responsiveness in all those individuals with limited mobility, to whom an active method would not be accessible.

**ADDITIONAL INFORMATION SECTION**

- The present study was funded by a dedicated grant (PSR Fondi Linea 2 -Tipologia A - 2016_CE) from the Department of Biomedical Sciences for Health, Università degli Studi di Milano
- The authors declared no professional relationship with companies or manufacturers that might benefit from the results of the present study.
- The authors declare that the results of the study are presented clearly, honestly, and without fabrication, falsification, or inappropriate data manipulation.
- The authors approved the final version of the manuscript.
- The authors agree to be accountable for all aspects of the work in ensuring that questions related to the accuracy or integrity of any part of the work are appropriately investigated and resolved
- All persons designated as authors qualify for authorship, and all those who qualify for authorship are listed.
- The data that support the findings of this study are available from the corresponding author upon reasonable request.

All experiments were conducted at the Physiology Labs of the School of Sport Science, Università degli Studi di Milano. All persons designated as authors qualify for authorship, and all those who qualify for authorship are listed.

- Conception or design of the study: EC, VM, FS, FE.
- Acquisition, analysis, or interpretation of data for the study: EC, MV, AVB, SL, AP, GC.
- Drafting the manuscript or revising it critically for important intellectual content: EC, MV, AVB, FS, FE.
- All the authors have approved the final version of the manuscript and agree to be accountable for all aspects of the study in ensuring that questions related to the accuracy or integrity of any part of the study are appropriately investigated and resolved.

**DECLARATIONS**

**Conflict of interest:** The Authors have no conflict of interest. The Authors are grateful to the participants that volunteered for the present investigation.

**Ethics approval:** The local University Ethics Committee approved the study (*CE* 27/17), which was performed in accordance with the principles of the latest version of the Declaration of Helsinki.

**Consent to participate**: All participants gave their written, informed consent after receiving an explanation of the study purpose and design. The participants were free to withdraw from the study at any time.

**Consent for publication:** All the participants gave their consent for data and study publication.

**Availability of data and material:** The data that support the findings of this study will be available at ClinicalTrial.gov (ID: NCT04758754) or from the corresponding author upon reasonable request.

**Acknowledgments:** The authors thank all participants of the study and all the examiners who helped in the clinical examinations and assessment procedures. The results of the study are presented clearly, honestly, and without fabrication, falsification, or inappropriate data manipulation, and statement that results of the present study do not constitute endorsement by ACSM.

**REFERENCES**

1. Nishiwaki M, Yonemura H, Kurobe K, Matsumoto N. Four weeks of regular static stretching reduces arterial stiffness in middle-aged men. *Springerplus*. 2015;4(555):1–11.

2. Qureshi G, Brown R, Salciccioli L, et al. Relationship between aortic atherosclerosis and non-invasive measures of arterial stiffness. *Atherosclerosis*. 2007;195:190–4.

3. Broxterman RM, Witman MA, Trinity JD, et al. Strong Relationship between Vascular Function in the Coronary and Brachial Arteries: A Clinical Coming of Age for the Updated Flow-Mediated Dilation Test? *Hypertension*. 2019;74(1):208–15.

4. Hotta K, Kamiya K, Shimizu R, et al. Stretching exercises enhance vascular endothelial function and improve peripheral circulation in patients with acute myocardial infarction. *Int Heart J*. 2013;54(2):59–63.

5. Puca AA, Carrizzo A, Villa F, et al. Vascular ageing: The role of oxidative stress. *Int J Biochem Cell Biol*. 2013;45(3):556–9.

6. Harvey A, Montezano AC, Touyz RM. Vascular biology of ageing-Implications in hypertension. *J Mol Cell Cardiol*. 2015;83(1):112–21.

7. Pedrinolla A, Colosio AL, Magliozzi R, et al. The Vascular Side of Chronic Bed Rest: When a Therapeutic Approach Becomes Deleterious. *J Clin Med*. 2020;9(4):918.

8. Venturelli M, Amann M, Layec G, et al. Passive leg movement-induced hyperaemia with a spinal cord lesion: Evidence of preserved vascular function. *Acta Physiol*. 2014;210(2):429–39.

9. Green DJ, Hopman MTE, Padilla J, Laughlin MH, Thijssen DHJ. Vascular Adaptation to Exercise in Humans: Role of Hemodynamic Stimuli. *Physiol Rev*. 2017;97(2):495–528.

10. Venturelli M, Amann M, Trinity JD, Ives SJ, Richardson RS. Spinal cord injury and vascular function: evidence from diameter-matched vessels. *J Appl Physiol*. 2021;130(3):562–70.

11. Kruse NT, Scheuermann BW. Cardiovascular Responses to Skeletal Muscle Stretching: “Stretching” the Truth or a New Exercise Paradigm for Cardiovascular Medicine? *Sport Med*. 2017;4(12):2507–20.

12. Spence AL, Carter HH, Naylor LH, Green DJ. A prospective randomized longitudinal study involving 6 months of endurance or resistance exercise. Conduit artery adaptation in humans. *J Physiol*. 2013;591(5):1265–75.

13. Kazmi RS, Boyce S, Lwaleed BA. Homeostasis of Hemostasis: The Role of Endothelium. *Semin Thromb Hemost*. 2015;41(6):549–55.

14. Davies KJA. Molecular Aspects of Medicine Adaptive homeostasis. *Mol Aspects Med*. 2016;49:1–7.

15. Birk G, Dawson E, Batterham A, et al. Effects of Exercise Intensity on Flow Mediated Dilation in Healthy Humans. *Int J Sports Med*. 2012;34(05):409–14.

16. Walsh K, Roberts J, Bennett G. Mobility in old age. *Gerodontology*. 1999;16(2):69–74.

17. Esposito F, Mathieu-Costello O, Shabetai R, Wagner PD, Richardson RS. Limited maximal exercise capacity in patients with chronic heart failure: Partitioning the contributors. *J Am Coll Cardiol*. 2010;55(18):1945–54.

18. Bisconti AV, Cè E, Longo S, et al. Evidence of improved vascular function in the arteries of trained but not untrained limbs after isolated knee-extension training. *Front Physiol*. 2019;12(10):1–12.

19. Wray DW, Witman MAH, Ives SJ, et al. Does brachial artery flow-mediated vasodilation provide a bioassay for NO? *Hypertension*. 2013;62(2):345–51.

20. Thijssen DHJ, Black MA, Pyke KE, et al. Assessment of flow-mediated dilation in humans: a methodological and physiological guideline. *Am J Physiol Circ Physiol*. 2011;300(1):H2–12.

21. Pyke KE, Tschakovsky ME. The relationship between shear stress and flow-mediated dilatation: implications for the assessment of endothelial function. *J Physiol*. 2005;568(2):357–69.

22. Wray DW, Uberoi A, Lawrenson L, Richardson RS. Evidence of preserved endothelial function and vascular plasticity with age. *Am J Physiol - Hear Circ Physiol*. 2006;290(3):H1271-1277.

23. Naylor LH, Carter H, FitzSimons MG, Cable NT, Thijssen DHJ, Green DJ. Repeated increases in blood flow, independent of exercise, enhance conduit artery vasodilator function in humans. *Am J Physiol - Hear Circ Physiol*. 2011;300(2):664–9.

24. Bisconti A V., Cè E, Longo S, et al. Evidence for improved systemic and local vascular function after long-term passive static stretching training of the musculoskeletal system. *J Physiol*. 2020;598(17):3645–66.

25. Wong A, Figueroa A. Eight weeks of stretching training reduces aortic wave reflection magnitude and blood pressure in obese postmenopausal women. *J Hum Hypertens*. 2014;28(4):246–50.

26. Niebauer J, Cooke JP. Cardiovascular effects of exercise: Role of endothelial shear stress. *J Am Coll Cardiol*. 1996;28(7):1652–60.

27. Green DJ, Bilsborough W, Naylor LH, et al. Comparison of forearm blood flow responses to incremental handgrip and cycle ergometer exercise: relative contribution of nitric oxide. *J Physiol*. 2005;562(2):617–28.

28. Minetto MA, Motta G, Gorji NE, et al. Reproducibility and validity of the Italian version of the International Physical Activity Questionnaire in obese and diabetic patients. *J Endocrinol Invest*. 2018;41(3):343–9.

29. Laginestra FG, Amann M, Kirmizi E, et al. Electrically induced quadriceps fatigue in the contralateral leg impairs ipsilateral knee extensors performance. *Am J Physiol Integr Comp Physiol*. 2021;320(5):R747–56.

30. Venturelli M, Layec G, Trinity J, Hart CR, Broxterman RM, Richardson RS. Single passive leg movement-induced hyperemia: a simple vascular function assessment without a chronotropic response. *J Appl Physiol*. 2017;122(1):28–37.

31. Trinity JD, Groot HJ, Layec G, et al. Nitric oxide and passive limb movement: a new approach to assess vascular function. *J Physiol*. 2012;590(6):1413–25.

32. Harris RA, Nishiyama SK, Wray DW, Richardson RS. Ultrasound assessment of flow-mediated dilation. *Hypertens (Dallas, Tex 1979)*. 2010;55(5):1075–85.

33. Venturelli M, Rampichini S, Coratella G, et al. Heart and musculoskeletal hemodynamic response to repetitive bouts of quadricepes static stretching. *J Appl Physiol*. 2019;127(2):376–84.

34. Cohen J. *Statistical power analysis for the behavioral sciences*. 2nd ed. Mahwah, NJ: Lawrence Erlbaum Associates; 1988.

35. Hopkins WG, Marshall SW, Batterham AM, Hanin J. Progressive statistics for studies in sports medicine and exercise science. *Med Sci Sports Exerc*. 2009;41(1):3–13.

36. Longo S, Cè E, Bisconti AV, et al. The effects of 12 weeks of static stretch training on the functional; mechanical; and architectural characteristics of the triceps surae muscle–tendon complex. *Eur J Appl Physiol*. 2021;121(6):1743–58.

37. Richardson RS, Leek BT, Gavin TP, et al. Reduced Mechanical Efficiency in Chronic Obstructive Pulmonary Disease but Normal Peak V̇o2 with Small Muscle Mass Exercise. *Am J Respir Crit Care Med*. 2004;169(1):89–96.

38. Esposito F, Reese V, Shabetai R, Wagner PD, Richardson RS. Isolated quadriceps training increases maximal exercise capacity in chronic heart failure: The role of skeletal muscle convective and diffusive oxygen transport. *J Am Coll Cardiol*. 2011;58(13):1353–62.

39. Esposito F, Mathieu-Costello O, Shabetai R, Wagner PD, Richardson RS. Limited maximal exercise capacity in patients with chronic heart failure: Partitioning the contributors. *J Am Coll Cardiol*. 2010;55(18):1945–54.

40. Holder SM, Bruno RM, Shkredova DA, et al. Reference Intervals for Brachial Artery Flow-Mediated Dilation and the Relation with Cardiovascular Risk Factors. *Hypertension*. 2021;77(5):1469–80.

41. Venturelli M, Cè E, Limonta E, et al. Central and peripheral responses to static and dynamic stretch of skeletal muscle: mechano- and metaboreflex implications. *J Appl Physiol*. 2017;122(1):112–20.

42. Gifford JR, Richardson RS. CORP: Ultrasound assessment of vascular function with the passive leg movement technique. *J Appl Physiol*. 2017;123(6):1708–20.

43. Cheng T-H, Chen JJ-W, Shih N-L, et al. Mechanical stretch induces endothelial nitric oxide synthase gene expression in neonatal rat cardiomyocytes. *Clin Exp Pharmacol Physiol*. 2009;36(5–6):559–66.

44. Jufri NF, Mohamedali A, Avolio A, Baker MS. Mechanical stretch: physiological and pathological implications for human vascular endothelial cells. *Vasc Cell*. 2015;7(1):8.

45. Trinity JD, Jonathan Groot H, Layec G, et al. Passive leg movement and nitric oxide-mediated vascular function: The impact of age. *Am J Physiol - Hear Circ Physiol*. 2015;308(6):H672–9.

46. Joyner MJ, Casey DP. Regulation of increased blood flow (hyperemia) to muscles during exercise: a hierarchy of competing physiological needs. *Physiol Rev*. 2015;95(2):549–601.

47. Poole DC, Mathieu-Costello O. Skeletal muscle capillary geometry: adaptation to chronic hypoxia. *Respir Physiol*. 1989;77(1):21–9.

48. Poole DC, Musch TI, Kindig CA. In vivo microvascular structural and functional consequences of muscle length changes. *Am J Physiol - Hear Circ Physiol*. 1997;272(5):41–5.

49. Trinity JD, Richardson RS. Physiological Impact and Clinical Relevance of Passive Exercise/Movement. *Sport Med*. 2019;49(9):1365–81.

50. Hellsten Y, Rufener N, Nielsen JJ, Høier B, Krustrup P, Bangsbo J. Passive leg movement enhances interstitial VEGF protein, endothelial cell proliferation, and eNOS mRNA content in human skeletal muscle. *Am J Physiol - Regul Integr Comp Physiol*. 2008;294(3):975–82.

**FIGURE CAPTIONS**

**Figure 1**. Study flow chart

**Figure 2.** Acute hemodynamic measurements.

Passive stretching bout. Acute passive stretching bout of the knee extensor muscles with the right arm maintaining actively the elongation and the left arm positioned on a table to allow the positioning of the finger photoplethysmography device without any constriction (Panel A), acute passive stretching bout the knee extensor muscles stretched by an operator (Panel B).

Single-leg knee extension. Exercise performed with (Panel C) and without (Panel D) he stabilization of the participant’s position on the ergometer.

**Figure 3.** Panels A and B: Photographs showing the passive stretching exercises. Each exercise includes a set of 5 stretches of 45 s with 15 s of rest in between. Panel A, hip extension + knee flexion in orthostatism, Panel B, hip extension + knee flexion position. Panels C1 and C2. Photographic representation of participant’s positioning on the knee extension ergometer.

**Figure 4.** Changes in femoral blood flow induced by single passive limb movement test before (PRE, open circle) and after (POST, closed circle) in single-leg knee extension training (SLKE, panel A, N = 12), passive static stretching training (PST, panel B, N = 12), and control group (CTRL, panel C, N = 12). Two-way mixed model ANOVA (time × groups); ANCOVA detected differences in changes between the groups. ^*^*P*<0.05 *vs.* baseline (PRE).

**Figure 5.** Individual percentage changes in flow-mediated dilation in percentage (%FMD) over shear rate, %FMD: flow-mediated dilation in percentage, and sum of shear rate after single-leg knee extension training (SLKE, N = 12), passive static stretching training (PST, N = 12), and control group (CTRL, N = 12). Box and whisker plot reports the median values together with the standard deviation, standard error, and the minimum-maximum range. Two-way mixed model ANOVA (time × groups); ANCOVA detected differences in changes between the groups. ^*^*P*<0.05 *vs.* baseline (Pre), ^c^*P*<0.05 vs CTRL.

**SUPPLEMENTAL DIGITAL CONTENT**

Supplemental digital content 1.docx—Characteristics of each single-leg knee extension training session
