# Supplementary material for: Long-Term Passive Leg Stretch Improves Systemic Vascular Responsiveness as Much as Single-Leg Exercise Training
Source: Med Sci Sports Exerc. 2021 Oct 25;54(3):475–88. doi: 10.1249/MSS.0000000000002811 (PMC10097495; doi:10.1249/MSS.0000000000002811)
Supplement: Supplementary file 2 [file msse-54-475-s002.docx]

Supplemental digital content 1. Characteristics of each single-leg knee extension training session.

| Single-Leg Knee Extension training | |
| --- | --- |
| Session | Duration and Workload (%Ẇmax) |
| Sessions 1 – 3 | 5 mins 70%Ẇmax  8 min; 75%  3 mins and 40 s 70%Ẇmax |
| Sessions 4 and 5 | 5 mins 70%Ẇmax  8 min; 80%  3 mins and 40 s 70%Ẇmax |
| Session 6 | New Ẇmax determination |
| Sessions 7 – 9 | 5 mins 70%Ẇmax  8 min; 80%  3 mins and 40 s 70% Ẇmax |
| Sessions 10 and 11 | 5 mins 70%Ẇmax  8 min; 85%  3 mins and 40 s 75% Ẇmax |
| Session 12 | New MWR determination |
| Sessions 13 –15 | 5 mins 70%Ẇmax  8 min; 85%  3 mins and 40 s 75%Ẇmax |
| Sessions 16 and 17 | 5 mins 70%Ẇmax  8 min; 90%  3 mins and 40 s 70%Ẇmax |
| Session 18 | New MWR determination |
| Sessions 19 – 21 | 5 mins 70%Ẇmax  8 min; 85%  3 mins and 40 s 75%Ẇmax |
| Sessions 22 and 23 | 5 mins 70%Ẇmax  8 min; 95%  3 mins and 40 s 70%Ẇmax |
| Session 24 | Post MWR determination |
